# THBS2 + cancer-associated fibroblasts promote EMT leading to oxaliplatin resistance via COL8A1-mediated PI3K/AKT activation in colorectal cancer

**DOI:** 10.1186/s12943-024-02180-y

**Published:** 2024-12-28

**Authors:** Xing Zhou, Jiashu Han, Anning Zuo, Yuhao Ba, Shutong Liu, Hui Xu, Yuyuan Zhang, Siyuan Weng, Zhaokai Zhou, Long Liu, Peng Luo, Quan Cheng, Chuhan Zhang, Yukang Chen, Dan Shan, Benyu Liu, Shuaixi Yang, Xinwei Han, Jinhai Deng, Zaoqu Liu

**Affiliations:** 1 Department of Interventional Radiology, The First Affiliated Hospital of Zhengzhou University, Zhengzhou, Henan 450052 China; 2 Interventional Institute of Zhengzhou University, Zhengzhou, Henan 450052 China; 3 Interventional Treatment and Clinical Research Center of Henan Province, Zhengzhou, Henan 450052 China; 4 Department of Pediatric Surgery, The First Affiliated Hospital of Zhengzhou University, Zhengzhou, Henan 450052 China; 5 Department of General Surgery, Peking Union Medical College Hospital, Beijing, 100020 China; 6 Department of Urology, The First Affiliated Hospital of Zhengzhou University, Zhengzhou, Henan 450052 China; 7 Department of Hepatobiliary Surgery, The First Affiliated Hospital of Xi’an Jiaotong University, Xi’an, Shaanxi 710061 China; 8 Department of Oncology, Zhujiang Hospital, Southern Medical University, Guangzhou, China; 9 Department of Neurosurgery, Xiangya Hospital, Central South University, Changsha, Hunan 410008 China; 10 Department of Oncology, The First Affiliated Hospital of Zhengzhou University, Zhengzhou, Henan 450052 China; 11 Clinical Science Institute, University Hospital Galway, Galway, Ireland; 12 Tianjian Laboratory of Advanced Biomedical Sciences, Academy of Medical Sciences, Zhengzhou University, Zhengzhou, China; 13 Department of Colorectal Surgery, The First Affiliated Hospital of Zhengzhou University, Zhengzhou University, Zhengzhou, China; 14 Richard Dimbleby Department of Cancer Research, Comprehensive Cancer Centre, Kings College London, London, UK; 15 Institute of Basic Medical Sciences, Chinese Academy of Medical Sciences and Peking Union Medical College, Beijing, 100730 China

**Keywords:** Colorectal cancer, Oxaliplatin resistance, Cancer-associated fibroblasts, COL8A1, EMT

## Abstract

Cancer-associated fibroblasts (CAFs) exert multiple tumor-promoting functions and are key contributors to drug resistance. The mechanisms by which specific subsets of CAFs facilitate oxaliplatin resistance in colorectal cancer (CRC) have not been fully explored. This study found that THBS2 is positively associated with CAF activation, epithelial-mesenchymal transition (EMT), and chemoresistance at the pan-cancer level. Together with single-cell RNA sequencing and spatial transcriptomics analyses, we identified THBS2 specifically derived from subsets of CAFs, termed THBS2 + CAFs, which could promote oxaliplatin resistance by interacting with malignant cells via the collagen pathway in CRC. Mechanistically, COL8A1 specifically secreted from THBS2 + CAFs directly interacts with the ITGB1 receptor on resistant malignant cells, activating the PI3K-AKT signaling pathway and promoting EMT, ultimately leading to oxaliplatin resistance in CRC. Moreover, elevated COL8A1 promotes EMT and contributes to CRC oxaliplatin resistance, which can be mitigated by ITGB1 knockdown or AKT inhibitor. Collectively, these results highlight the crucial role of THBS2 + CAFs in promoting oxaliplatin resistance of CRC by activating EMT and provide a rationale for a novel strategy to overcome oxaliplatin resistance in CRC.

**Supplementary Information:**

The online version contains supplementary material available at 10.1186/s12943-024-02180-y.

## Introduction

Colorectal cancer (CRC) is the third most common cancer worldwide, with an annual incidence exceeding 1.9 million cases [1]. Beyond surgical intervention, chemotherapy remains a cornerstone in CRC management, aiming to shrink tumors and prevent future growth and spread [2]. Oxaliplatin, a platinum-based chemotherapeutic agent, is extensively utilized in the treatment of various cancers, notably CRC, ovarian cancer (OV), gastric cancer (STAD), and pancreatic cancer (PAAD) [3–6]. Oxaliplatin together with extra chemotherapeutic agents, such as fluorouracil and/or irinotecan, constitute FOLFOX or FOLFIRINOX protocols, which are approved as first-line treatment for advanced and metastatic CRC [7, 8]. Tragically, drug resistance remains a significant global challenge in the clinical management of CRC with oxaliplatin-based chemotherapy [9, 10]. Therefore, it is fundamentally important to figure out effective ways to overcome this resistance and enhance treatment outcomes.

In the tumor microenvironment (TME), various cells crosstalk with each other is critical for cancer progression. Cancer-associated fibroblasts (CAFs), a principal component of the TME, constitute a heterogeneous group of stromal cells with diverse origins, phenotypes, functions, and abundances across various cancer types [11]. CAFs have a far-reaching impact on tumor-promoting functions and play crucial roles in drug resistance through multiple mechanisms, such as extracellular matrix (ECM) remodeling and the promotion of epithelial-mesenchymal transition (EMT), highlighting their potential values as prognostic factors and therapeutic targets [12–14]. More importantly, contrary to the general belief that CAFs invariably promote tumor progression, targeting CAFs has been shown to exacerbate the disease in PAAD and mouse models [15, 16], implying that different CAFs subsets may perform opposing roles in disease progression. Therefore, precisely identifying the cancer-promoting CAFs subsets requires the discovery of specific biomarkers to distinguish CAFs subpopulations and to understand their activities and mechanisms. Recent studies have demonstrated that cell-cell interactions between CAFs and malignant cells give rise to chemotherapy resistance in various cancer types, including CRC [17]. Nevertheless, the mechanisms of action of the CAFs subgroups correlated with oxaliplatin resistance in CRC have not yet been fully elucidated.

Here, by integrating with multi-omics data, we provided evidence that THBS2 derived from specific subsets of CAFs, defined as THBS2 + CAFs, correlated with dismal prognosis and EMT activity across various cancer types. Functionally, THBS2 + CAFs remarkably positively correlated with the aggressive phenotype and oxaliplatin resistance in CRC. Single-cell RNA sequencing (scRNA-seq) and spatial transcriptomics (ST) revealed that THBS2 + CAFs had more interactions and closer distance with resistant cells, respectively. Mechanistically, COL8A1, specifically secreted from THBS2 + CAFs, directly interacted with the ITGB1 receptor expressed on malignant cells, thereby activating EMT and promoting oxaliplatin resistance via the phosphatidylinositol 3-kinase (PI3K)-AKT pathway. Additionally, in vitro and in vivo experiments confirmed that COL8A1 contributed to cancer progression and resistance in CRC, which could be mitigated by ITGB1 knockdown or AKT inhibitor. Therefore, our study uncovered the crucial role of THBS2 + CAFs in oxaliplatin resistance and highlighted its potential as a predictive biomarker and therapeutic target to overcome oxaliplatin resistance in CRC.

## Materials and methods

### Pan-cancer bulk expression and phenotype data collection

The pan-cancer multi-omics, encompassing transcriptomic, copy number variations (CNV), and corresponding clinical information, from The Cancer Genome Atlas (TCGA) and Genotype-Tissue Expression (GTEx) were collected from UCSC Xena (https://xenabrowser.net/datapages/). The single nucleotide variant (SNV) and methylation data were obtained from Genomic Data Commons (GDC) (https://gdc.cancer.gov/) [18]. Additionally, four tumor microenvironment (TME) subtypes, six immune subtypes, and the TCGA subtypes of OV and head and neck squamous cell carcinoma (HNSC) were downloaded from the corresponding literature [19]. The consensus molecular subtypes (CMS) of CRC were defined by the CMScaller R package [20].

### Pan-cancer protein data collection

The Clinical Proteomic Tumor Analysis Consortium (CPTAC) contained more than 1,000 untreated primary samples across 10 cancer types, including matched normal samples. LinkedOmicsKB (https://kb.linkedomics.org/) [21] allows to more readily download CPTAC data, including protein expression data, clinical information, and phenotype data. Among others, there were no normal samples for breast invasive carcinoma (BRCA) and glioblastoma multiforme (GBM).

### Collection and analysis of expression data and drug sensitivity data of pan-cancer cell lines

Pan-cancer cell line expression data and cell line annotations were downloaded from the Cancer Cell Line Encyclopedia (CCLE). The drug sensitivity data of cancer cell lines were collected from Genomics of Drug Sensitivity in Cancer (GDSC) and Cancer Therapeutics Response Portal (CTRP). The gene expression profile data for cell lines and their corresponding drug sensitivity values could be downloaded from https://osf.io/temyk. The pRRophetic R package [22] was applied to predict the chemotherapeutic response by integrating TCGA expression data, cancer cell line expression data and corresponding drug sensitivity data of cell lines. Simultaneously, CellMiner (http://discover.nci.nih.gov/cellminer/) [23] also offered two processed files, including mRNA expression and drug data, to research the correlation between expression and pharmacological data for cell lines. In GDSC, CRC cell lines were categorized as oxaliplatin-resistant or sensitive groups based on upper and lower one-quarter thresholds of oxaliplatin drug sensitivity value to predict the similarity between TCGA-CRC and cell line expression.

### Pan-cancer scRNA-seq and ST data collection and preparation

The scRNA-seq data, encompassing CRC-EMTAB8107, BRCA-EMTAB8107, OV-EMTAB8107, PAAD-CRA001160, and HNSC-GSE103322, were obtained from the TISCH website (http://tisch.comp-genomics.org/) [24]. The Seurat R package [25] was implemented to construct a Seurat object from the gene expression matrices. Cells with 500 ~ 4,000 UMI/cell, 500 ~ 6,000 genes/cell, and < 10% mitochondrial genes were retained. Batch effect correction was executed utilizing the harmony R package [26]. We performed principal component analysis (PCA) to reduce the dimensionality of scRNA-seq data and selected the top 30 principal components (PCs) for uniform manifold approximation and projection (UMAP). Cell clusters were identified using the FindClusters function and annotated based on typical markers of different cell types collected from literature [27–29]. The inferCNV R package [30] was adopted to distinguish malignant and non-malignant cells with the parameters: “denoise”, default hidden Markov model (HMM) settings, cutoff = 1 for Smart-seq2 or cutoff = 0.1 for 10x Genomics. The T, NK, B, and myeloid cells served as a normal reference. We conducted the FindAllMarkers and FindMarkers function to identify differentially expressed genes (DEGs).

The ST data of CRC were obtained from http://www.cancerdiversity.asia/scCRLM/ [31] and https://www.10xgenomics.com/. The ST data of OV and BRCA were obtained from https://www.10xgenomics.com/. Reanalyzed publicly available ST data of PAAD and HNSC can be accessed from the GEO database under accession codes: GSE203612 and GSE181300, respectively [32, 33]. The publicly available ST datasets were loaded into a Seurat object by the Seurat R package. Subsequently, low-quality spots with gene counts below 300 and mitochondrial gene counts exceeding 30% were filtered out.

### Statistics and reproducibility

Public data processing, visualization, and statistical analysis were carried out utilizing the R 4.3.0 software. Spearman’s correlation coefficient was applied to estimate the correlation between two continuous variables. For continuous variables, differences between two groups or among over two groups were examined utilizing the Wilcoxon rank sum test or the Kruskal-Wallis test. For categorical variables, the Chi-square test was employed. False discovery rate (FDR) was applied to adjust the *p*-values. All *p*-values were two-sided. To confirm the results, we carried out the experiments in multiple replicates to confirm their reproducibility.

Additional methods applied in this study were available in Supplementary Methods.

## Results

### THBS gene family was generally upregulated and significantly associated with disease progression in pan-cancer

**Fig. 1:**
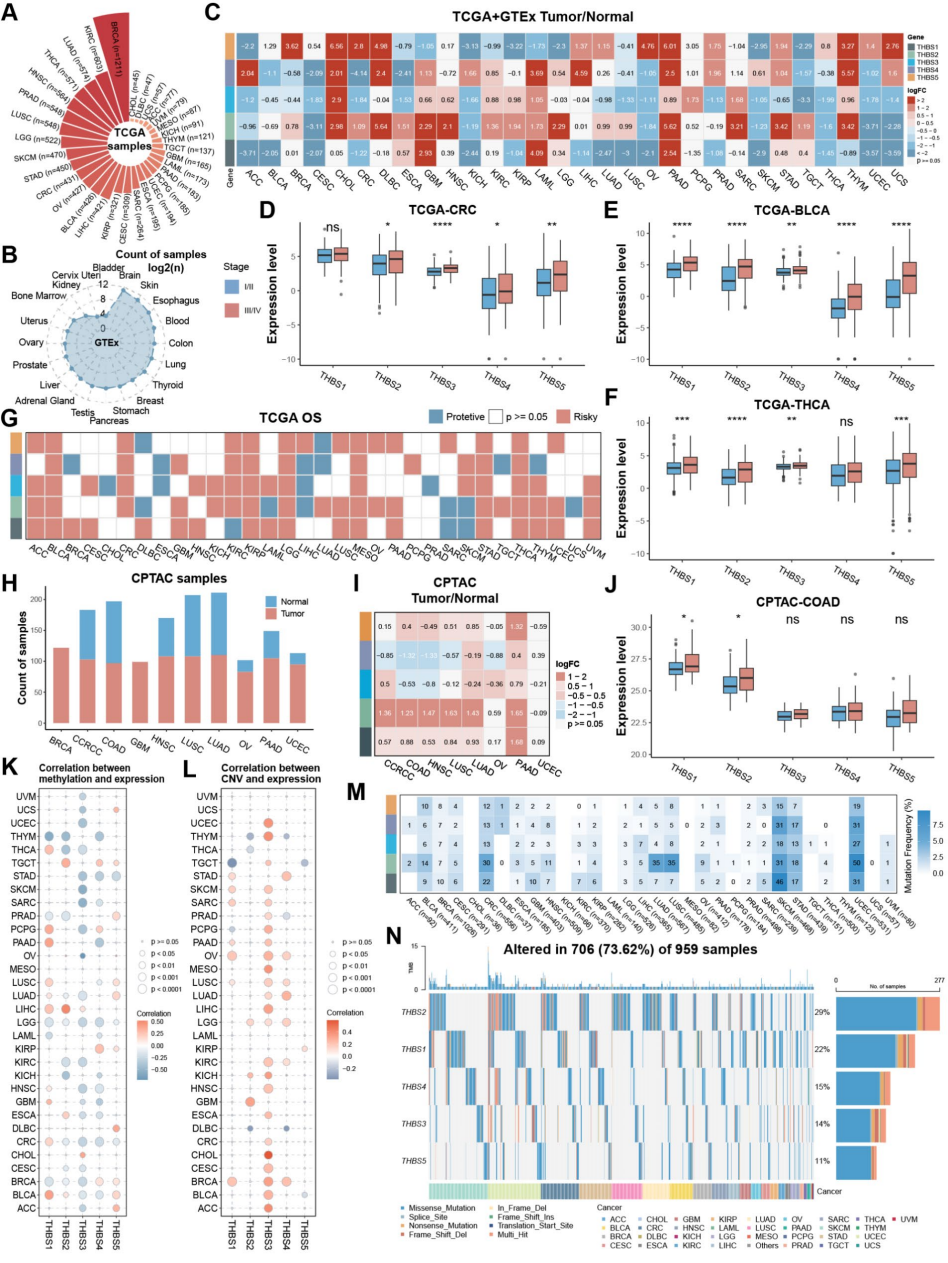
THBS gene family is generally upregulated and significantly associated with disease progression in pan-cancer. (**A**-**B**) The number of TCGA and GTEx samples applied in this study, respectively. (**C**) The mRNA expression differences between normal and tumor samples. (**D**-**F**) The mRNA expression differences in distinct stages of CRC, BLCA, and THCA, respectively. (**G**) The correlation of THBS family genes and OS in TCGA using the log-rank test. Genes with *p* < 0.05 and HR > 1 were considered risky, while those with *p* < 0.05 and HR < 1 were considered protective. (**H**) The number of normal and tumor samples in CPTAC applied in this study. (**I**) The protein expression differences between normal and cancer samples. (**J**) The protein expression differences in distinct stages of COAD. (**K**-**L**) The Spearman correlation between mRNA expression and methylation and CNV, respectively. Blue points represent negative correlation and red points represent positive correlation. (**M**) Mutation frequency of THBS family genes in 32 cancers. Numbers represent the number of samples with the corresponding mutated gene for a given cancer. ‘0’ indicates no mutation in the gene coding region, and the absence of a number indicates no mutation in any region of the gene. (**N**) SNV oncoplot showing the mutation distribution of THBS family genes and the classification of SNV types. ns, not significant, *p < 0.05, **p < 0.01, ***p < 0.001, ****p < 0.0001

To evaluate the expression difference and prognostic significance of THBS genes in pan-cancer, this study collected cancer sample data from multiple databases, encompassing transcriptomic, proteomic, and cell lines data (Fig. 1A-B). As illustrated in Fig. 1C, THBS mRNA expression levels varied in cancer types. Compared to normal tissues, THBS1 and THBS2 showed higher expression in brain cancers and PAAD, but lower expression in cervical squamous cell carcinoma and endocervical adenocarcinoma (CESC), OV, uterine corpus endometrial carcinoma (UCEC) and uterine carcinosarcoma (UCS). THBS3 and THBS4 expression exhibited the same trend in most cancer types, albeit with variable degrees of significance in cancer types. THBS2 and THBS5 presented significantly different between tumor and normal samples in nearly all cancer types, with THBS2 being significantly higher in kidney cancers and brain cancers compared to normal tissues, while THBS5 exhibited the opposite trend. Of note, both THBS2 and THBS5 had particularly high expression in lymphoid neoplasm diffuse large B-cell lymphoma (DLBC), thymoma (THYM) and gastrointestinal tumors, including cholangiocarcinoma (CHOL), CRC, PAAD, and STAD. Additionally, these genes showed higher expression in stage III/IV compared to stage I/II in several cancer types, including CRC, bladder urothelial carcinoma (BLCA), and thyroid carcinoma (THCA) (Fig. 1D-F). Most THBS family genes were associated with poor overall survival (OS) in cancers (Fig. 1G). In six cancers, BLCA, kidney renal papillary cell carcinoma (KIRP), lower grade glioma (LGG), mesothelioma (MESO), STAD, and THCA, all genes were associated with dismal prognosis. However, some genes were linked to favorable prognoses in specific cancers, suggesting discrepancies in the impact of individual genes on cancer outcomes in distinct cancer types. Subsequently, the protein data was utilized to investigate the relationship between gene expression and clinical outcomes at the protein level (Fig. 1H). THBS1, THBS2 and THBS5 proteins exhibited relatively high expression in six cancer types compared to normal samples, except for THBS5 in clear cell renal cell carcinoma (CCRCC), while THBS3 and THBS4 proteins showed lower levels in colon adenocarcinoma (COAD) and HNSC (Fig. 1I). For UCEC, THBS gene family proteins were not notably different. Moreover, THBS1 and THBS2 protein levels showed slightly higher in stage III/IV compared to stage I/II in COAD (Fig. 1J).

Next, the methylation profiles of THBS family genes were portrayed. Interestingly, the methylation level of THBS3 was generally inversely correlated with its mRNA level, whereas others did not exhibit a significant correlation in most cancers (Fig. 1K). Copy number variants (CNVs) level of THBS3 was significantly positively correlated with its mRNA level in 24 of 32 cancer types (Fig. 1L). Additionally, single nucleotide polymorphism (SNP) data was summarized to profile the frequency and variant types in each cancer (Fig. 1M-N). UCEC, skin cutaneous melanoma (SKCM), and CRC had high single nucleotide variant (SNV) frequencies ranging from 12% to 50%. The frequency of THBS2 was beyond 30% in CRC, lung adenocarcinoma (LUAD), lung squamous cell carcinoma (LUSC), SKCM, and UCEC. THBS2 had the highest mutation rate, followed by THBS1, THBS4, THBS3, and THBS5, with mutation percentages of 29%, 22%, 15%, 14% and 11%, respectively. In summary, most THBS family genes were upregulated in cancer samples and high stages and were associated with poor outcomes, with a weaker correlation with genomic alterations.

### THBS2 facilitated the CAF activation and EMT phenotype

**Fig. 2:**
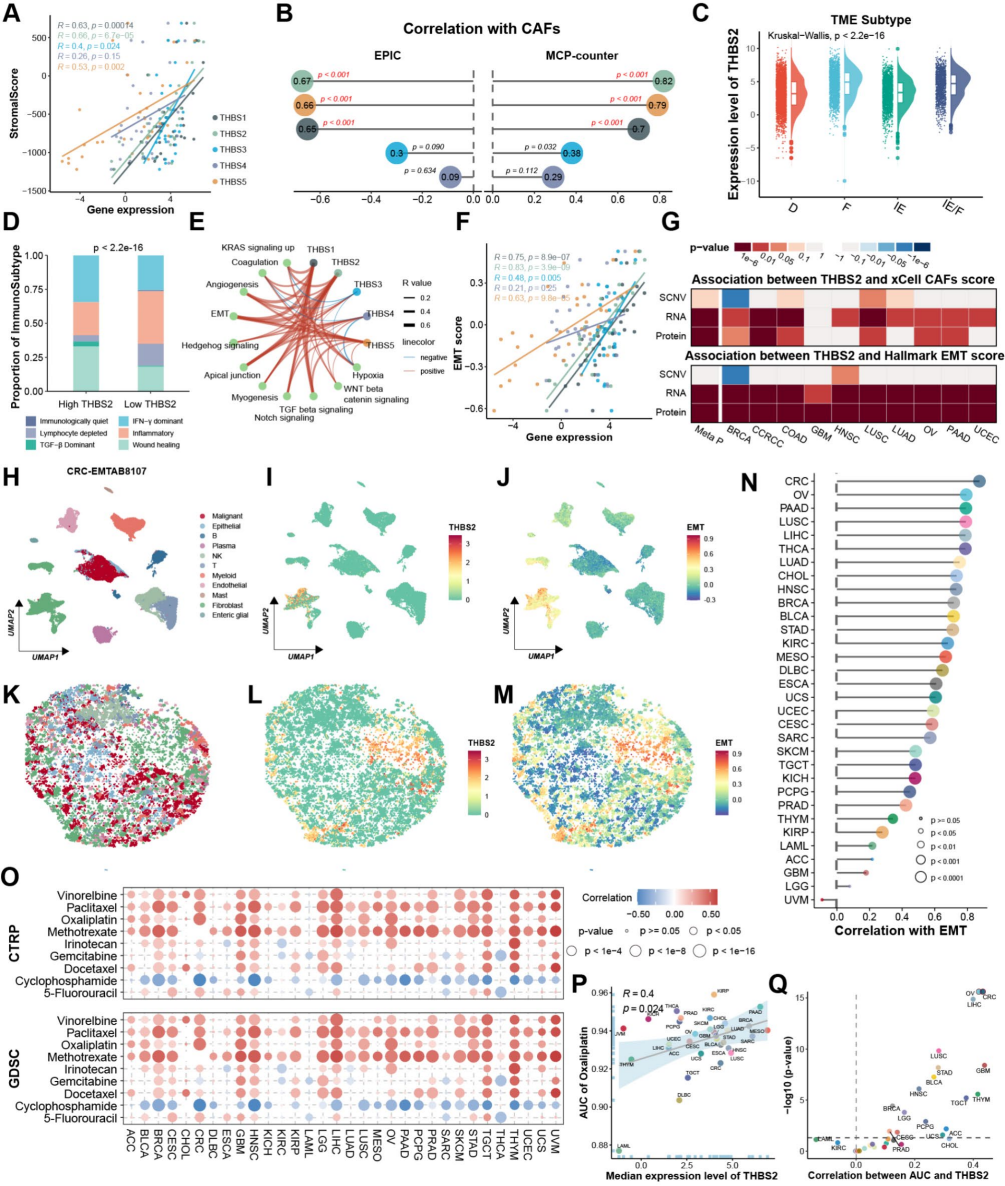
THBS2 facilitated CAF activation and EMT phenotype. (**A**) The Spearman correlation of THBS family mRNA expression and stromal score calculated by ESTIMATE algorithm in TCGA. (**B**) The Spearman correlation of THBS family mRNA expression and CAFs abundance deconvoluted by EPIC and MCP-counter algorithms in TCGA. (**C**) The difference in THBS2 mRNA expression among distinct TME subtypes derived from Bagaev et al. (**D**) The six immune subtypes derived from Bagaev et al. distribution in the high-THBS2 group and low-THBS2 group. The Chi-square test was used and the samples were divided into a high-THBS2 group and a low-THBS2 group based on the median THBS2 mRNA expression. (**E**) The Spearman correlation of THBS family mRNA expression and Hallmark pathway activity calculated by the gene set variation analysis (GSVA) algorithm in TCGA. (**F**) The Pearson correlation of THBS family mRNA expression and EMT score calculated by GSVA algorithm in TCGA. (**G**) The Spearman correlation of THBS2 protein expression and CAFs and EMT scores derived from the CPTAC database. (**H**) UMAP plot of the identified cell types. Different colors represented the different cell types. (**I**-**J**) UMAP plot of the identified cells colored by THBS2 expression and the EMT score calculated by AddMouduleScore function, respectively. (**K**) Assignment of cell subtypes and their spatial distributions inferred by the CellTrek algorithm in CRC ST sample. (**L**-**M**) The expression of THBS2 and EMT score calculated by AddMouduleScore function based on ST of CRC, respectively. (**N**) The Spearman correlation of THBS2 mRNA expression and EMT score calculated by GSVA algorithm for each cancer type in TCGA. (**O**) The Spearman correlation of THBS2 mRNA expression and drug AUC derived from GDSC and CTRP for each cancer type in TCGA. The chemotherapeutic drug AUC values for each sample were predicted by pRRophetic algorithm. (**P**) The Spearman correlation of median THBS2 mRNA expression and median AUC of oxaliplatin across the cancer types in TCGA. The oxaliplatin AUC value for each sample was predicted by pRRophetic algorithm. (**Q**) The Spearman correlation of THBS2 mRNA expression and AUC of oxaliplatin for each cancer type. The oxaliplatin AUC value for each sample was predicted by pRRophetic algorithm. Points colored by red represented *p* < 2.2e-16

Furthermore, THBS genes exhibited low expression in several cell lines, such as CRC, STAD, PAAD, BRCA, and OV, suggesting that these genes are predominantly expressed in non-malignant cells of the TME (Figure S1A-E). Consistently, THBS family genes were positively correlated with the stromal score, especially THBS2, with no or weak correlation with the immune score or tumor purity (Fig. 2A and FigureS1F-G). Subsequently, correlation with immune and stromal cells defined by the MCP-counter and EPIC algorithms [34, 35] revealed that CAF population was most dramatically associated with THBS2 (Fig. 2B and Figure S1H). Meanwhile, TME classification as proposed by Bagaev et al. [19] displayed that THBS2 varied among four subtypes, with immune-enriched/fibrotic (IE/F) and fibrotic (F) characterized by CAF activation, TGF-β pathway and EMT transition, having the highest THBS2 expression (Fig. 2C). Recent studies have proposed multiple molecular subtyping systems to profile complex heterogeneity, such as the consensus molecular subtypes (CMS) of CRC [36]. THBS2 expression was the highest in the CMS4 of CRC, mesenchymal subtypes of OV, and HNSC, with CMS4 and mesenchymal subtype characterized by EMT upregulation [36–38] (Figure S1**I-K)**. Consistently, high THBS2 levels were more prevalent in the TGF-β dominant subtype associated with immunosuppressed TME [39] (Fig. 2D). To further elucidate their potential function in cancer progression, the correlation between THBS genes and Hallmark pathway activity was analyzed. The results revealed a positive correlation with EMT, particularly notable for THBS2 (Fig. 2E-F). Similar patterns were discovered at the protein level, reinforcing the consistency and reliability of THBS2 associated with CAFs and EMT (Fig. 2G).

Simultaneously, multiple scRNA-seq datasets revealed that THBS2 was predominantly expressed in CAFs (Fig. 2H-I). CAFs displayed the highest EMT score, suggesting the strongest correlation with EMT among all cell types (Fig. 2J and Figure S2). Furthermore, leveraging the CellTrek tool [40], the scRNA-seq datasets were mapped to corresponding ST data to decipher their spatial organization to visualize expression levels on a spatial scale. Consistent with our single-cell findings, CAFs exhibited the highest THBS2 expression and EMT score (Fig. 2K-M and Figure S2).

Notably, the correlation between THBS2 expression and EMT activity varied in cancers (Fig. 2N). CRC showed the strongest, followed by OV and PAAD, both of which had abundant CAFs in the TME [41, 42]. Given that activated CAFs and EMT could facilitate drug resistance [14, 43], the connection of THBS2 with the area under the curve (AUC) values of the drugs applied in clinical practice was analyzed. These results exhibited that THBS2 had a remarkable positive correlation with the drug AUC in BRCA, CRC, HNSC, and STAD, suggesting THBS2 could serve as a biomarker for chemoresistance (Fig. 2O). Specifically, THBS2 was positively correlated with the AUC of oxaliplatin, especially in CRC, implying it might contribute to CRC resistance to oxaliplatin (Fig. 2P-Q). However, 5-fluorouracil and irinotecan, commonly used chemotherapeutic agents for CRC treatment [44], showed no or weak correlation with THBS2 (Figure S1L-O). Collectively, these results suggested that THBS2 was correlated with the CAFs, EMT, and chemoresistance, and potentially facilitated oxaliplatin resistance in CRC.

### THBS2 + CAFs were associated with oxaliplatin resistance in CRC

**Fig. 3:**
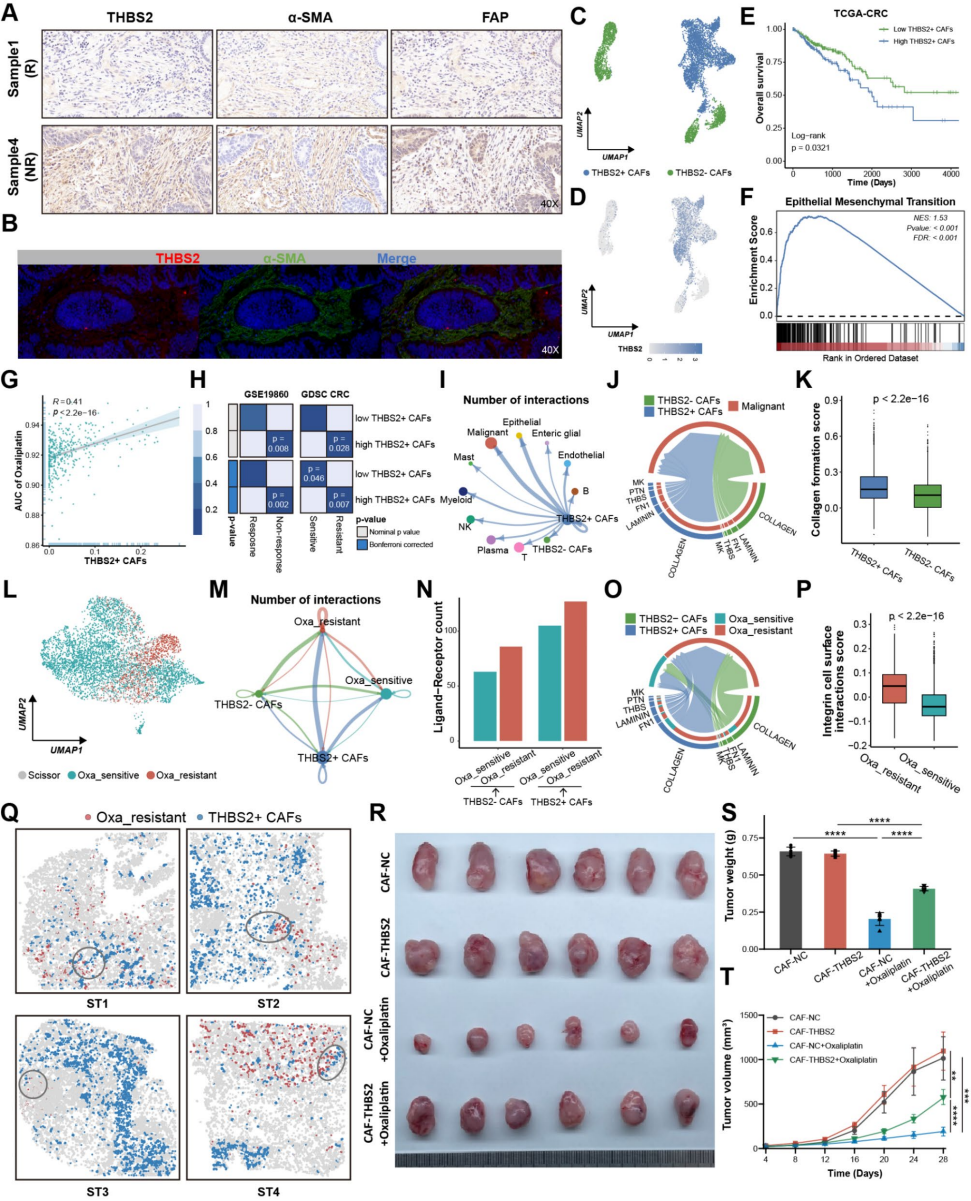
THBS2 + CAFs were associated with oxaliplatin resistance in CRC. (**A**) Representative images of IHC staining for THBS2, α-SMA and FAP in responder (R) group and non-responder (NR) group. (**B**) Representative images of mIF for THBS2 and α-SMA. (**C**) UMAP plot of the distinct CAFs subtypes identified by THBS2 expression level. Different colors represented the different subtypes. (**D**) UMAP plot showing THBS2 expression across different CAFs subtypes. (**E**) Kaplan–Meier curve of OS between high and low THBS2 + CAFs abundance deconvoluted by CIBERSORTx algorithm in TGCA-CRC. (**F**) Gene set enrichment analysis (GSEA) analysis showing EMT pathway was upregulated in high THBS2 + CAFs abundance subgroup. (**G**) The Spearman correlation of THBS2 + CAFs abundance and AUC of oxaliplatin in TCGA-CRC. The oxaliplatin AUC value for each sample in TCGA-CRC was predicted by pRRophetic algorithm. (**H**) SubMap algorithm evaluated the expression similarity and the chemotherapy response between TCGA-CRC and GSE19860 treated with mFOLFOX6 and GDSC CRC cell lines treated with oxaliplatin. (**I**) Number of interactions from THBS2 + CAFs to other cells inferred by CellChat algorithm. (**J**) Chord diagram showing the cell-cell interaction pathways among THBS2 + CAFs, THBS2- CAFs, and malignant cells inferred by CellChat algorithm. (**K**) The difference in collagen formation score calculated by AddMouduleScore function between THBS2 + CAFs and THBS2- CAFs. (**L**) UMAP plot of the distinct malignant cell subtypes identified via the Scissor algorithm. Different colors represented the different subtypes. (**M**) The counts of ligand-receptor interactions from CAFs subtypes to malignant cell subtypes inferred by CellChat algorithm. (**N**) Number of interactions from CAFs subtypes to malignant cell subtypes inferred by CellChat algorithm. (**O**) Chord diagram showing the cell-cell interaction pathways among CAFs subtypes and malignant cell subtypes inferred by CellChat algorithm. (**P**) The difference in integrin cell surface interaction score calculated by AddMouduleScore function between the resistant and sensitive subtype. (**Q**) Spatial cell charting of resistant malignant cells and THBS2 + CAFs using CellTrek algorithm. (**R**-**T**) Representative images of subcutaneous xenografts from SW480 cells mixed with CAF-THBS2 or combined with oxaliplatin treatment. Tumor weight and tumor volume were measured after implantation. **p < 0.01, ***p < 0.001, ****p < 0.0001

The immunohistochemistry (IHC) and multiplex immunofluorescence (mIF) of pre-treatment human CRC samples further validated that THBS2 was predominantly located at the CAFs areas and consistently higher in non-responders, irrespective of chemotherapy application, suggesting that THBS2 may contribute to primary therapeutic resistance (Fig. 3A-B and Figure S3A-C). Meanwhile, THBS2 failed to correlate with CRC mutation profiles, such as KRAS, BRAS and microsatellite status, as well as anatomical location (Figure S3D-G). Subsequently, we re-categorized the CAFs into THBS2 + CAFs and THBS2- CAFs to illustrate the impact of THBS2 + CAFs on malignant cells. THBS2 was predominantly expressed in CAFs subclusters 0, 1, 2, 7, and 8, and thus these clusters were termed THBS2 + CAFs, while the remaining clusters were labeled as THBS2- CAFs (Fig. 3C-D and Figure S4A-B). Firstly, deconvolution of scRNA-seq of CRC utilizing the CIBERSORTx algorithm [45] revealed that patients with a higher proportion of THBS2 + CAFs had shorter OS, disease-free survival (DSS), and progression-free survival (PFS), and were highly enriched in the EMT pathway (Fig. 3E-F and Figure S4C-D). Additionally, THBS2 + CAFs abundance was positively associated with the AUC of oxaliplatin (Fig. 3G). A subsequent analysis utilizing Subclass mapping (SubMap) [46] confirmed these above results, indicating that THBS2 + CAFs facilitated oxaliplatin resistance (Fig. 3H). The reciprocal interactions between THBS2 + CAFs and the TME further displayed that THBS2 + CAFs exhibited more interactions with malignant cells, primarily involving ECM, including collagen, laminin, FN1, and THBS pathways (Fig. 3I-J and Figure S4E-F). Notably, the collagen pathway constituted the major part of these interactions and enriched highly in THBS2 + CAFs, implying the collagen pathway served a fundamental role in chemotherapy resistance (Fig. 3J-K).

Using the oxaliplatin-based chemotherapy RNA-seq data containing 20 non-responders and 9 responders, we conducted the Scissor algorithm [47] to define the resistant malignant cells (Fig. 3L). The Beyondcell [48] and pRRophetic [22], drug sensitivity prediction tools at the single-cell and bulk level, respectively, collectively corroborated the accuracy and reliability of Scissor in defining resistant cells, which had a lower Beyondcell score (BCS) and higher AUC value of oxaliplatin (Figure S4G-J). Resistant cells exhibited more communications with THBS2 + CAFs, which were predominantly the collagen pathway (Fig. 3M-O and Figure S4K). Meanwhile, resistant cells had a high integrin cell surface interaction score, implying resistant cells had more collagen receptors involved in interacting with THBS2 + CAFs (Fig. 3P). ST further revealed that THBS2 + CAFs were closer to resistant cells on a spatial scale (Fig. 3Q and Figure S4L-M). It is noteworthy that ST3 and ST4 showed fewer interactions and more distance between THBS2 + CAFs and resistant cells, which may be attributed to the fact that they were from patients with partial response (PR) after neoadjuvant chemotherapy [31]. Furthermore, we explored the effects of THBS2 + CAFs on CRC resistance in subcutaneous xenograft model and Thbs2 conditional knockout mouse model. Initially, primary CAFs were isolated from fresh CRC tissues and stably transfected with THBS2 (CAF-THBS2) using lentiviral transduction. Then, each mouse received a subcutaneous injection into the right axilla of a mixture containing CRC cells combined with CAF-NC or CAF-THBS2 cells to subcutaneous xenograft model. For fibroblast-specific Thbs2 knockout mice (Thbs2^flox/flox^; Col1a2-CreER), wildtype and floxed alleles of the Thbs2 gene are targeted by Cre recombinase. Western blotting validated that THBS2 was stably overexpressed or knocked out in CAF-THBS2 and Thbs2 knockout mice (CKO), respectively (Figure S5A-B). In vivo experiments showed that CAF-THBS2 group had significantly larger tumor volume and weight than CAF-NC, while the CKO group had significantly smaller volume and weight than control (Thbs2^flox/flox^) (Fig. 3R-T and Figure S5C-D). Collectively, THBS2 + CAFs exhibited extensive interactions with malignant cells and facilitated CRC oxaliplatin resistance.

### COL8A1 derived from THBS2 + CAFs facilitated oxaliplatin resistance in CRC

**Fig. 4:**
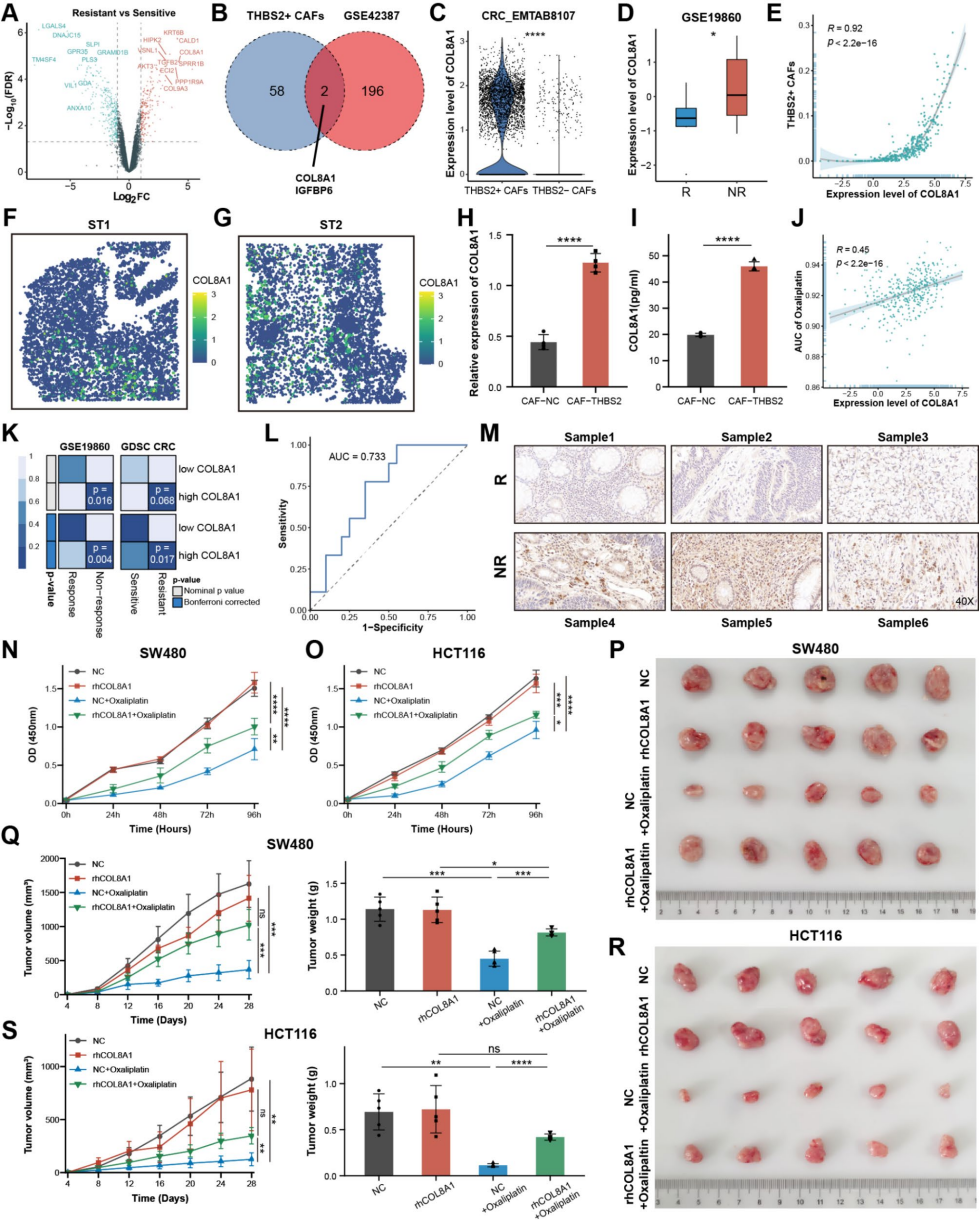
COL8A1 derived from THBS2 + CAFs facilitated oxaliplatin resistance in CRC. (**A**) Volcano plot showing the DEGs between resistant and sensitive cells in GSE42387. Those with log2FC > 1 and FDR < 0.05 were considered upregulated colored by red, while those with log2FC < -1 and FDR < 0.05 were considered downregulated colored by blue. (**B**) Venn-diagram intersected THBS2 + CAFs marker genes calculated by FindMarkers function and upregulated DEGs in oxaliplatin-resistant CRC cells. Genes with FDR < 0.05 and log2FC > 1 in THBS2 + CAFs were considered marker genes. (**C**) Violin plot showing COL8A1 expression level across different CAF subtypes. (**D**) The difference in COL8A1 expression between non-response (NR) and response (R) group in GSE19860. (**E**) The Spearman correlation of COL8A1 expression and THBS2 + CAFs abundance deconvoluted by CIBERSORTx algorithm in TCGA-CRC. (**F**-**G**) The expression level of COL8A1 based on ST analysis. (**H**) qPCR analysis of COL8A1 mRNA levels in CAF-THBS2 and CAF-NC. (**I**) ELISA quantification of COL8A1 levels in the supernatant of CAF-THBS2 and CAF-NC cultures. (**J**) The Spearman correlation of COL8A1 expression and AUC of oxaliplatin in TCGA-CRC. The oxaliplatin AUC value for each sample in TCGA-CRC was predicted by pRRophetic algorithm. (**K**) SubMap algorithm evaluated the expression similarity and the chemotherapy response between TCGA-CRC and GSE19860 treated with mFOLFOX6, and GDSC CRC cell lines treated with oxaliplatin. (**L**) ROC curve showing COL8A1 expression level predicting response efficiency to oxaliplatin-based chemotherapy. (**M**) Representative images of IHC staining for COL8A1 in responder (R) and non-responder (NR) groups. (**N**-**O**) The CCK8 assay compared the proliferation rates of CRC cells treated with oxaliplatin alone or combined with rhCOL8A1. (**P**-**Q**) Representative images of subcutaneous xenografts from SW480 cells treated with rhCOL8A1 alone or combined with oxaliplatin. Tumor weight and tumor volume were measured after implantation. (**R**-**S**) Representative images of subcutaneous xenografts from HCT116 cells treated with rhCOL8A1 alone or combined with oxaliplatin. Tumor weight and tumor volume were measured after implantation. ns, not significant, *p < 0.05, **p < 0.01, ***p < 0.001, ****p < 0.0001

To provide further insight into the underlying resistance mechanism, the differential gene analysis between oxaliplatin-resistant and sensitive CRC cells was conducted [49] and displayed that the resistant cells exhibited up-regulated genes involved in ECM and collagen pathways, such as COL8A1, COL9A3, and TGFB2 (Fig. 4A and Figure S6A). In particular, COL8A1 and IGFBP6 were shared by the upregulated DEGs in resistant cells and THBS2 + CAFs marker genes, suggesting they might contribute to resistance (Fig. 4B). Following in-depth analysis, COL8A1 exhibited specific expression in THBS2 + CAFs and significantly higher expression in the non-responder group, while IGFBP6 did not (Fig. 4C-D and Figure S6B-C). Additionally, COL8A1 RNA and protein levels were positively correlated with CAFs abundance, particularly THBS2 + CAFs (Fig. 4E and Figure S6D-F). ST further revealed COL8A1 was highly expressed in the THBS2 + CAFs areas (Fig. 4F-G and Figure S6G-H). qPCR and ELISA confirmed that COL8A1 exhibited remarkable higher expression in CAF-THBS2, while remarkable lower expression level in CAFs from the fibroblast-specific Thbs2 knockout mice (Fig. 4H-I and Figure S6I-J). Meanwhile, we wondered whether COL8A1 could be secreted by CRC cells leading to drug resistance by autocrine fashion. qPCR and ELISA detected the level of COL8A1 in different CRC cell lines [50–52]. Compared to CAF-THBS2, COL8A1 was poorly expressed or even not expressed in CRC cells, which was supported by scRNA-seq analysis showing COL8A1 derived from THBS2 + CAFs rather than malignant cells (Figure S6K-M). Therefore, these findings revealed that COL8A1 was mainly secreted from THBS2 + CAFs.

To fully illustrate whether COL8A1 could facilitate oxaliplatin resistance, further analyses from multiple standpoints were carried out. Firstly, transcriptomic analysis found that COL8A1 was strongly correlated with the AUC of oxaliplatin and resistance group in CRC (Fig. 4J-K). The area under the receiver operating characteristic (ROC) curve indicated that the powerful ability of COL8A1 expression level predicted oxaliplatin-based chemotherapy resistance (Fig. 4L). Next, IHC staining of CRC human samples validated that COL8A1 was highly expressed in non-responders (Fig. 4M). Then, CCK8 assays further revealed that oxaliplatin could inhibit CRC cells, while the inhibitory effect was antagonized by elevating COL8A1 (Fig. 4N-O). Lastly, subcutaneous xenografts models were established by subcutaneously implanting CRC cells and treated with oxaliplatin alone or combined with recombinant human COL8A1 (rhCOL8A1). Oxaliplatin significantly reduced tumor weight and volume in the xenograft models, however, the combination with rhCOL8A1 antagonized the anti-tumor effort (Fig. 4P-S). In summary, these data indicated that COL8A1 could facilitate oxaliplatin resistance in CRC.

### COL8A1 activated EMT leading to oxaliplatin resistance in CRC

**Fig. 5:**
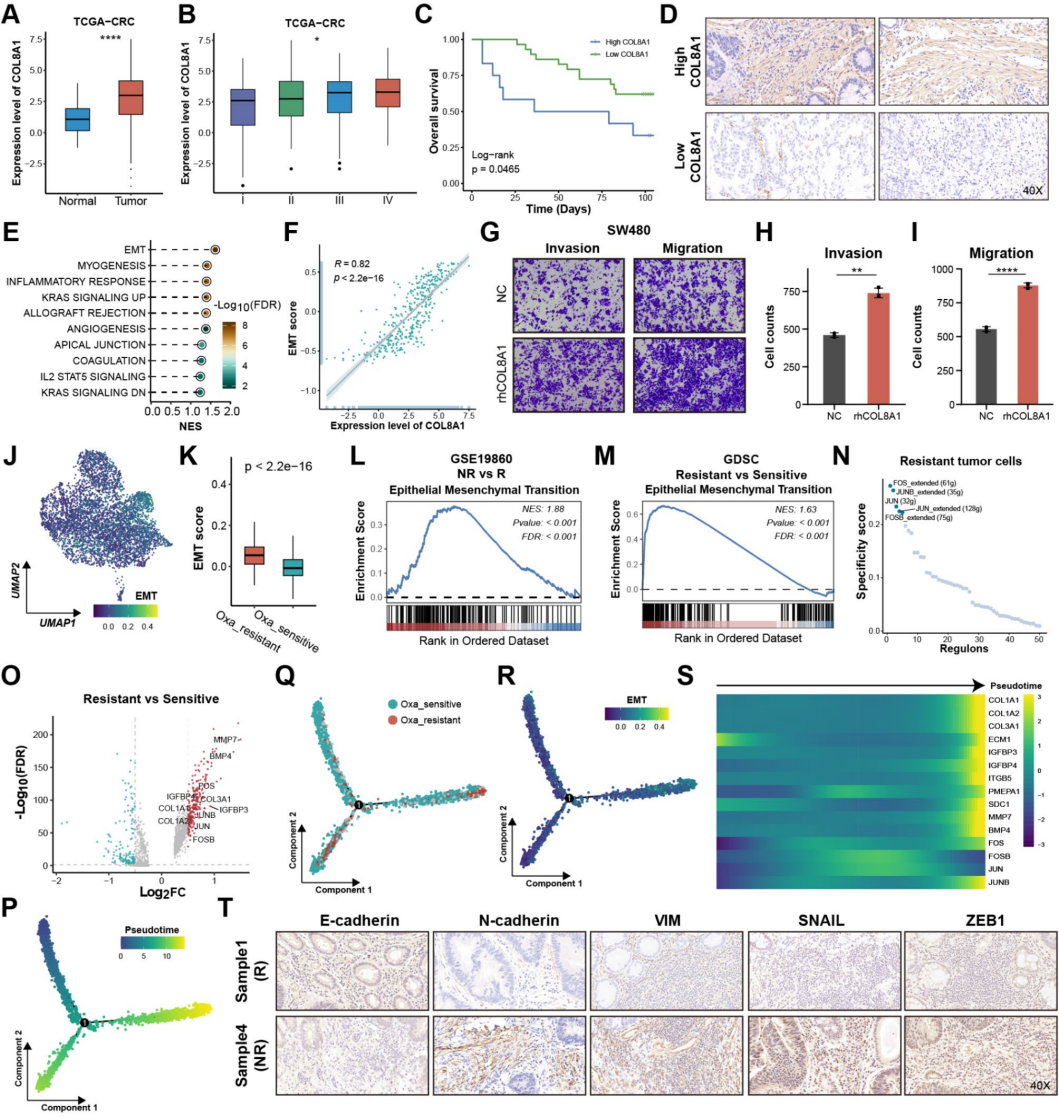
COL8A1 activated EMT leading to oxaliplatin resistance in CRC. (**A**) The difference in COL8A1 expression between tumor and normal samples in TCGA-CRC. (**B**) The difference in COL8A1 expression across distinct stages in TCGA-CRC. (**C**) Kaplan-Meier curve of OS between high and low COL8A1 expression using clinical samples. (**D**) Representative images of IHC staining for COL8A1 in high COL8A1 group and low COL8A1 group. (**E**) GSEA showing pathway activity in high and low COL8A1 groups in TCGA-CRC. (**F**) The Spearman correlation of COL8A1 expression and EMT score calculated by GSVA in TCGA-CRC. (**G**-**I**) Representative data from invasion and migration assays performed in SW480 cells treated with rhCOL8A1 or NC. (**J**) UMAP plot of EMT score calculated by AddMouduleScore function in malignant cells. (**K**) The difference in EMT score calculated by AddMouduleScore function between oxaliplatin-resistant and sensitive cells at the single cell level. (**L**-**M**) GSEA showing the EMT signaling pathway was upregulated in the NR group of GSE19860 and oxaliplatin-resistant CRC cell lines of GDSC, respectively. (**N**) Identification of activated TFs in oxaliplatin-resistant cells using the SCENIC algorithm. (**O**) DEGs between oxaliplatin-resistant and sensitive cells at the single-cell level. Genes with FDR < 0.05 were considered significant. Those with log2FC > 0.5 were considered upregulated genes colored by red, while Those with log2FC < -0.5 were considered downregulated genes colored by blue. (**P**-**R**) Temporal analysis of the acquired resistance in malignant cells colored by Pseudotime, malignant cell subclusters, and EMT score using Monocle 2 algorithm. (**S**) Temporal increase in the expression of TFs and other genes associated with the EMT using Monocle 2 algorithm. (**T**) Representative images of IHC staining for E-cadherin, N-cadherin, VIM, ZEB1 and SNAIL in responder (R) group and non-responder (NR) group. *p < 0.05, **p < 0.01, ***p < 0.001, ****p < 0.0001

Transcriptome analysis further revealed that COL8A1 was highly expressed in tumor samples and stage III/IV, as well as closely correlated with shorter survival time, underscoring its highly malignant behavior (Fig. 5A-B and Figure S7A), which was validated by clinical CRC samples (Fig. 5C-D). Gene set enrichment analysis (GSEA) revealed that high COL8A1 group showed higher EMT activity and its RNA and protein levels were remarkably associated with the EMT score (Fig. 5E-F and Figure S7B). EMT exerts a crucial function in regulating the invasive and migratory abilities of malignant cells [53]. As expected, exposure to rhCOL8A1 resulted in a marked increase in the invasive and migratory capabilities of CRC cells, while no difference was observed in cell proliferation (Fig. 5G-I and Figure S7C-G). Notably, scRNA-seq analysis found that the resistant cells had higher EMT score than sensitive cells (Fig. 5J-K). GSEA of oxaliplatin-based chemotherapy RNA-seq and GDSC CRC cell lines data revealed that non-responders and resistant CRC cell lines had higher EMT activity, underscoring that EMT may facilitate CRC oxaliplatin resistance (Fig. 5L-M). Considering the pivotal role of transcription factors (TFs) in regulating gene expression, SCENIC decoded the uniquely activated TFs in oxaliplatin-resistant cells to uncover the mechanism [54]. The JUN family and FOS family activities were up-regulated in the resistant cells (Fig. 5N and Figure S8A), which had been proven to regulate E-cadherin (CDH1), N-cadherin (CDH2), and SNAIL2 expression in cancers [55–57]. Concomitantly, differential expression analysis at the single-cell level revealed that the JUN and FOS gene expression levels were up-regulated in resistant cells (Fig. 5O and Figure S8B). To discover the process of switching from sensitive to resistant cells, Monocle 2 R package [58] was conducted to explore the pseudo-temporal developmental trajectory. The oxaliplatin-resistant cells occupied the terminal phase of the trajectory (Fig. 5P-Q). Further analysis revealed that the gradual generation of EMT phenotype during transition, and the expression of identified EMT TFs and markers exhibited a progressively increasing trend (Fig. 5R-S). Meanwhile, COL8A1 showed a remarkable positive correlation with EMT markers, except CDH1, implying COL8A1 may regulate their expression to promote EMT (Figure S8C). Consistently, IHC staining of clinical samples further displayed that non-responder group had higher expression level of N-cadherin, VIM, ZEB1 and SNAIL, while lower level of E-cadherin (Fig. 5T and Figure S8D). Briefly, COL8A1 may activate the EMT phenotype, thereby leading to oxaliplatin resistance in CRC.

### COL8A1 interacting with ITGB1 contributed to oxaliplatin resistance

**Fig. 6:**
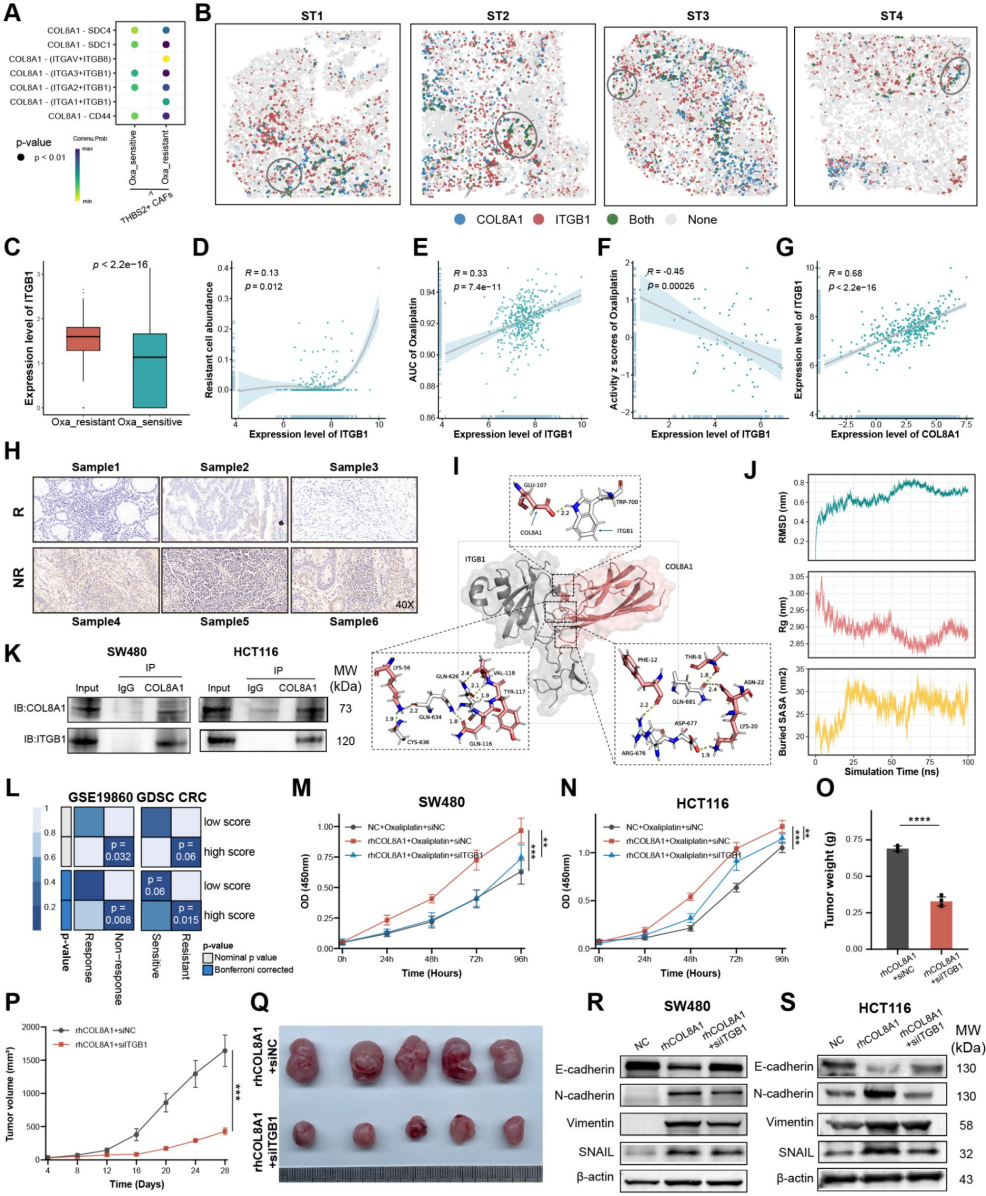
COL8A1 interacting with ITGB1 contributed to oxaliplatin resistance. (**A**) Interactions between THBS2 + CAFs and resistant malignant cells inferred by CellChat algorithm. (**B**) Visualization of COL8A1-ITGB1 interaction for ST data. (**C**) The difference in ITGB1 expression between resistant and sensitive malignant cells at the single-cell level. (**D**-**E**) The Spearman correlation of ITGB1 expression and resistant cell abundance deconvoluted by CIBERSORTx and AUC of oxaliplatin in TCGA-CRC, respectively. The oxaliplatin AUC value for each sample in TCGA-CRC was predicted by pRRophetic algorithm. (**F**) The Spearman correlation of ITGB1 expression and activity of oxaliplatin in CellMiner database. (**G**) The Spearman correlation of COL8A1 expression and ITGB1 expression in TCGA-CRC. (**H**) Representative images of IHC staining for ITGB1 in responder (R) group and non-responder (NR) group. (**I**) Molecular dynamics simulation of the COL8A1-ITGB1 complex, with structural visualization of key interacting residues. (**J**) Quantitative analysis of the COL8A1-ITGB1 complex stability over a 100-nanosecond simulation, showing Root Mean Square Deviation (RMSD), Radius of Gyration (Rg), and Buried Surface Area (SASA) values. (**K**) Co-IP demonstrated the protein interaction between COL8A1 and ITGB1 in CRC cells. (**L**) SubMap algorithm evaluated the similarity and the chemotherapy response between TCGA-CRC and GSE19860 treated with mFOLFOX6, and GDSC CRC cell lines treated with oxaliplatin. COL8A1-ITGB1 score was calculated based on the average ligand and receptor expression. (**M**-**N**) The CCK8 assay compared the proliferation rates of CRC cells treated with oxaliplatin plus rhCOL8A1 or combined with siITGB1. (**O**-**Q**) Representative images of subcutaneous xenografts from CRC cells treated with rhCOL8A1 alone or combined with oxaliplatin. Tumor weight and tumor volume were measured after implantation. (**R**-**S**) Western blotting showing EMT markers levels in CRC cells treated with rhCOL8A1 or combined with siITGB1. **p < 0.01, ***p < 0.001, ****p < 0.0001

To gain more insight into the biological functions of how COL8A1 derived from THBS2 + CAFs facilitating oxaliplatin resistance, CellChat program was applied to analyze and identify specific cell-cell interaction (CCI) [59]. CCI displayed THBS2 + CAFs exerted more interactions with resistant malignant cells via COL8A1-ITGB1 (Fig. 6A). The ST revealed that COL8A1 had more colocalization with ITGB1 than ITGB8, thereby ITGB1 may play a particularly vital role in communication (Fig. 6B and Figure S9A). Meanwhile, ITGB1 was highly expressed in resistant cells, positively correlated with resistant cells abundance, oxaliplatin AUC value, and COL8A1 expression, and negatively correlated with oxaliplatin activity, further indicating the potential impact of ITGB1 on resistance (Fig. 6C-G). Notably, IHC validated that ITGB1 showed higher expression level in non-responder group (Fig. 6H), indicating that ITGB1 may act as a crucial role in resistance.

To investigate the potential interaction ability of the COL8A1-ITGB1, we conducted molecular docking analysis to confirm the binding activity between COL8A1 and ITGB1. Structural visualization revealed specific interactions at the amino acid level contribute to the complex stability of COL8A1-ITGB1 complex (Fig. 6I). The stability of the COL8A1-ITGB1 complex was supported by quantitative metrics over a 100-nanosecond simulation (Fig. 6J). The Root Mean Square Deviation (RMSD) showed a rapid initial rise followed by a stabilization, indicating that the complex achieved a stable conformation early in the simulation. Additionally, the Radius of Gyration (Rg) and Buried Surface Area (SASA) analyses confirmed a compact and stable interaction interface between COL8A1 and ITGB1 (Fig. 6J). These results collectively demonstrate the robust binding affinity and structural integrity of the COL8A1-ITGB1 complex. Moreover, experimental Co-IP confirmed that COL8A1 could directly interact with ITGB1, thereby collectively leading to the development of resistance (Fig. 6K). Likewise, SubMap also revealed patients with high COL8A1-ITGB1 score were more likely to be non-sensitive to chemotherapy (Fig. 6L). To experimentally validate our observations, in vitro culture of CRC cells with COL8A1 or siITGB1 revealed siITGB1 could weaken the effect of rhCOL8A1 in facilitating oxaliplatin resistance (Fig. 6M-N). Consistently, in in vivo experiment, adding siITGB1 could significantly reduce tumor volume and weight (Fig. 6O-Q). Moreover, wound healing and transwell discovered the migratory and invasive capabilities of CRC cells declined after exposure to siITGB1 (Figure S9B-I). Western blotting validated that elevated COL8A1 could upregulate N-cadherin, SNAIL, and Vimentin, and downregulate E-cadherin, while combination with siITGB1 reduced EMT markers levels (Fig. 6R-S). Together, these results suggested the COL8A1 could directly interact with malignant cells via COL8A1-ITGB1, which stimulated malignant cells to acquire resistance to oxaliplatin.

### COL8A1 interfered with PI3K-AKT signaling promoting oxaliplatin resistance

**Fig. 7:**
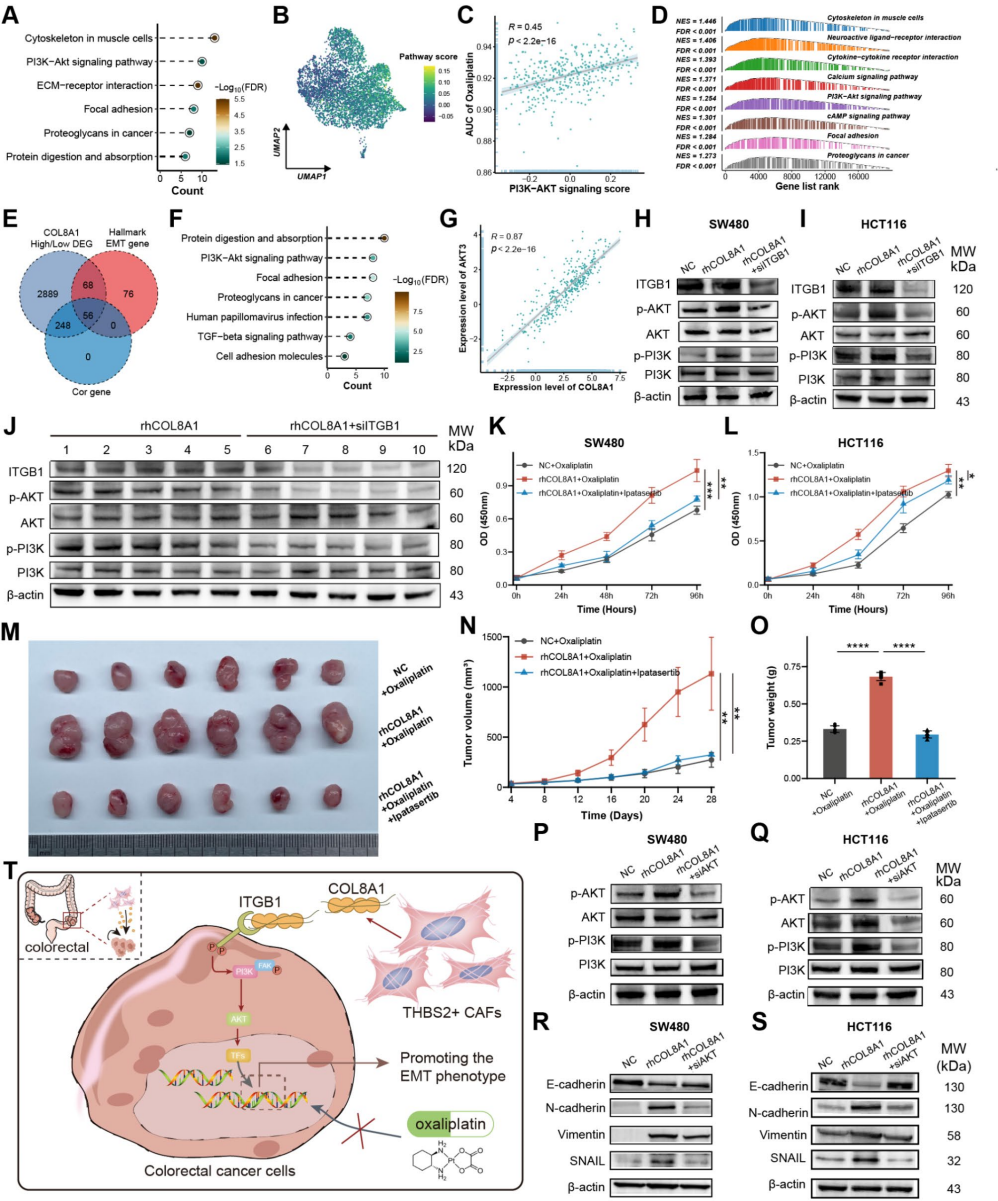
COL8A1 interfered with PI3K-AKT signaling promoting oxaliplatin resistance. (**A**) The KEGG analysis of DEGs between malignant resistant cells and sensitive cells calculated by FindMarkers function at the single-cell level. (**B**) UMAP plot of PI3K-AKT pathway score calculated by AddMouduleScore function in malignant cells. (**C**) The Spearman correlation of PI3K-AKT pathway score calculated by GSVA algorithm and AUC of oxaliplatin in TCGA-CRC. The oxaliplatin AUC value for each sample in TCGA-CRC was predicted by the pRRophetic algorithm. (**D**) GSEA showing PI3K-AKT pathway was upregulated in the high COL8A1 group of TCGA-CRC. (**E**) Venn-diagram intersected DEGs between high and low COL8A1 group, Hallmark EMT genes and genes correlated with COL8A1 greater than 0.8. (**F**) The KEGG analysis of 56 intersected genes. (**G**) The Spearman correlation of AKT3 expression and COL8A1 expression in TCGA-CRC. (**H**-**I**) Western blotting showing PI3K-AKT pathway activity in CRC cells treated with rhCOL8A1 or combined with siITGB1. (**J**) Western blotting showing PI3K-AKT pathway activity treated with rhCOL8A1 or combined with siITGB1 in subcutaneous xenograft mouse model. (**K**-**L**) The CCK8 assay compared the proliferation rates of CRC cells treated with oxaliplatin plus rhCOL8A1 or combined with AKT inhibitor Ipatasertib. (**M**-**O**) Representative images of subcutaneous xenografts from SW480 cells treated with rhCOL8A1 alone or combined with AKT inhibitor Ipatasertib. Tumor weight and tumor volume were measured after implantation. (**P**-**Q**) Western blotting showing PI3K-AKT pathway activity in CRC cells treated with rhCOL8A1 or combined with siAKT. (**R**-**S**) Western blotting showing EMT markers levels in CRC cells treated with rhCOL8A1 or combined with siAKT. (**T**) Schematic representation of the suggested mechanism by which COL8A1 mediates cancer cell survival and tumor progression

For a deeper mechanistic understanding, KEGG analysis of DEGs between resistant and sensitive malignant cells at the single-cell level revealed resistant cells highly enriched in the PI3K-AKT pathway (Fig. 7A-B). The PI3K-AKT pathway score exhibited a positive correlation with the AUC of oxaliplatin, suggesting that PI3K-AKT played a potential role in oxaliplatin resistance (Fig. 7C). Additionally, GSEA displayed patients with high COL8A1 exhibited activated PI3K-AKT activity and the shared gene significantly enriched in the PI3K-AKT pathway (Fig. 7D-F). COL8A1 was robustly positively correlated with PI3K-AKT pathway score and downstream gene expression (Fig. 7G and Figure S10A-B). These results provided us insight into the possibility that COL8A1 may potentially activate the PI3K-AKT leading to oxaliplatin resistance. These in silico findings were supported by Western blotting, which indicated elevated expression of p-AKT upon rhCOL8A1, while deceased level of p-AKT upon siITGB1 (Fig. 7H-I). Similarly, Western blotting also showed that rhCOL8A1 activated PI3K but could be antagonized by siITGB in the mice (Fig. 7J).

Furthermore, Beyondcell was employed to screen out potential compounds for oxaliplatin-resistant cells [48]. Four PI3K-AKT signaling inhibitors, tabelisib, KU-0063794, AZD8055, and BYL-719 were observed, suggesting PI3K-AKT inhibitors could mitigate oxaliplatin resistance (Figure S10C-D). In vitro AKT inhibitor Ipatasertib was used to confirm the drug-resistant effect of the PI3K-AKT pathway. The CCK8 and subcutaneous xenograft mouse model displayed that Ipatasertib could reverse the contribution of COL8A1 to oxaliplatin resistance and remarkably reduce tumor volume and weight (Fig. 7K-O). The migratory and invasive capabilities of CRC cells declined after treatment with Ipatasertib (Figure S10E-L). Western blotting confirmed that PI3K-AKT pathway and EMT phenotype were reduced after exposure to siAKT in CRC cells (Fig. 7P-S). Together, these results proposed that COL8A1 directly interacting with ITGB1 could activate the PI3K-AKT pathway and promote EMT, thereby leading to CRC resistance to oxaliplatin (Fig. 7T).

## Discussion

Although oxaliplatin-based chemotherapy is a standard treatment approach for CRC, the management of oxaliplatin resistance remains an ongoing obstacle [3, 60]. Comprehending the interplay between cancer cells and TME components was pivotal for devising effective cancer treatment strategies [11]. Mounting evidence highlights highly heterogeneous CAFs play crucial roles in cancer progression and therapy resistance [14]. However, the specific CAFs subgroups facilitating CRC oxaliplatin resistance remain elusive, thus underscoring the necessity for further investigation into CAFs heterogeneity. In this study, we comprehensively and thoroughly investigated the transcriptome characterizations of THBS family genes at the pan-cancer level, indicating that THBS2 was positively associated with CAFs, EMT, and chemoresistance. Together with scRNA-seq and ST, this study identified THBS2 + CAFs that could lead to EMT activation and oxaliplatin resistance via the collagen pathway in CRC. Importantly, our study uncovered a previously unrecognized mechanism whereby COL8A1 secreted from THBS2 + CAFs binds to ITGB1 expressed on resistant cells, leading to the PI3K-AKT and EMT activation, thereby leading to oxaliplatin resistance. Inhibition of COL8A1 attenuated cancer progression and enhanced oxaliplatin sensitivity, highlighting the promising role of COL8A1 as a target for overcoming oxaliplatin resistance.

By mining multi-omics profiling data, we first depicted the expression, methylation, and mutation profiles of THBS family genes. These results revealed that gene expression was the predominant responsible for malignant behaviors and cancer progression, rather than genetic and epigenetic alteration. THBS family genes are mainly expressed in stromal cells, especially in CAFs. In particular, THBS2 was remarkably positively correlated with CAF activation and EMT at the pan-cancer level, confirmed by scRNA-seq and ST. THBS2 has been reported to act as a diagnostic and prognostic biomarker and be associated with cancer progression and recurrence in several cancer types [61–65]. Meanwhile, we discovered the high expression of THBS2 strongly positively correlated with the AUC values of chemotherapy agents, thereby, THBS2 might serve as a biomarker predicting chemoresistance [66, 67].

A previous study found that high THBS2 + CAFs displayed a poor response to immunotherapy in LUAD [65], however, the potential impact of THBS2 + CAFs on clinical chemotherapy has not yet been unveiled. In this study, we integrated multi-omics data and observed that THBS2 + CAFs abundance was adversely correlated with chemotherapy response. Moreover, THBS2 + CAFs were closer to resistant malignant cells in spatial distance and had more interactions with resistant cells, suggesting that THBS2 + CAFs facilitated CRC oxaliplatin resistance. The collagen pathway played a crucial role in communications between THBS2 + CAFs and resistant cells, which has been reported to have an important impact on controlling cancer growth, progression, and therapeutic response [68]. A collagen ligand, COL8A1, specifically secreted from THBS2 + CAFs facilitated oxaliplatin resistance in CRC and could serve as a biomarker predicting chemoresistance. ITGB1, one of the most common subunits in the integrin family, is widely overexpressed in cancers and plays a non-negligible role in mediating resistance to diverse anti-cancer drugs [69–72]. ITGB1, coupled with distinct integrin α subunits, serves as the receptor for a wide variety of collagens, like collagen I-IV, VI, and X [69]. A recent study found paracrine and autocrine COL8A1 could bind to ITGB1 promoting tumor progression and gemcitabine resistance in PAAD [73]. Consistently, COL8A1 and ITGB1 showed spatial co-localizing and directly interact with each other in our study, suggesting they together contributed to the development of oxaliplatin resistance.

The amount of data gathered showed that chemotherapeutic resistance development is related to the EMT process [43]. Consistent with previous studies, we revealed that EMT was involved in oxaliplatin resistance at bulk and single-cell levels. COL8A1 exhibited a strong association with the EMT phenotype and promoted invasion and migration, while ITGB1 knockdown attenuated the EMT phenotype, suggesting targeting the ligand-receptor interaction could be a potential novel strategy. Additionally, JUN family and FOS family TFs were up-regulated in oxaliplatin-resistant cells, and gradually elevated expression during the transition from sensitive cells to resistant cells. JUN family and FOS family TFs have been reported to facilitate the EMT process by regulating E-cadherin, N-cadherin, and SNAI2 expression in cancers [55–57]. Moreover, the PI3K-AKT pathway was previously implicated in CRC progression and chemoresistance [74, 75], which is demonstrated by our findings. These findings are compatible with the idea that the PI3K-AKT pathway might trigger the EMT process by downregulating epithelial markers, while upregulating mesenchymal markers and EMT-specific transcription factors, thereby promoting CRC invasion, migration, and resistance [76, 77]. Notably, we further discovered some PI3K-AKT inhibitors might be effective for oxaliplatin-resistant cells. In combination with chemotherapy, Ipatasertib, a pan-AKT inhibitor, could enhance anti-tumor activity and improve survival time in multiple cancers [78, 79]. These data suggested that a combination with PI3K-AKT inhibitors might overcome the mechanism of oxaliplatin resistance in CRC.

In conclusion, this study found that COL8A1 derived from THBS2 + CAFs interacting with ITGB1 enhances EMT activity via the PI3K-AKT pathway leading to oxaliplatin resistance in CRC.

## Electronic supplementary material

Below is the link to the electronic supplementary material.

Supplementary Material 1

Supplementary Material 2

Supplementary Material 3

## Data Availability

All published data used in this work can be acquired from public databases. Other data used for this study are available from the corresponding author upon reasonable request.

## Abbreviations

AUC: Area under the curve
BCS: Beyondcell score
CAFs: Cancer-associated fibroblasts
CCI: Cell-cell interaction
CCLE: Cancer Cell Line Encyclopedia
CMS: Consensus molecular subtypes
CNVs: Copy number variants
CPTAC: Clinical Proteomic Tumor Analysis Consortium
CRC: Colorectal cancer
CTRP: Cancer Therapeutics Response Portal
DEGs: Differentially expressed genes
DSS: Disease-free survival
ECM: Extracellular matrix
EMT: Epithelial mesenchymal transition
FDR: False discovery rate
GDSC: Genomics of Drug Sensitivity in Cancer
GSEA: Gene set enrichment analysis
GSVA: Gene set variation analysis
GTEx: Genotype-Tissue Expression
IHC: Immunohistochemistry
KEGG: Kyoto Encyclopedia of Genes and Genomes
Log2FC: Log2 fold change
mIF: Multiplex immunofluorescence
OS: Overall survival
PFS: Progression-free survival
PI3K: Phosphatidylinositol 3-kinase
rhCOL8A1: Recombinant human COL8A1
scRNA-seq: Single-cell RNA sequencing
SNP: Single nucleotide polymorphism
SNV: Single nucleotide variant
ST: Spatial transcriptomics
SubMap: Subclass mapping
TCGA: The Cancer Genome Atlas
TME: Tumor microenvironment
