# Supplementary material for: THBS2 + cancer-associated fibroblasts promote EMT leading to oxaliplatin resistance via COL8A1-mediated PI3K/AKT activation in colorectal cancer
Source: Mol Cancer. 2024 Dec 28;23:282. doi: 10.1186/s12943-024-02180-y (PMC11681647; doi:10.1186/s12943-024-02180-y)
Supplement: Supplementary file 1 — Supplementary Material 1 [file 12943_2024_2180_MOESM1_ESM.docx]

**Supplementary Information for**

**THBS2+ Cancer-Associated Fibroblasts Promote EMT Leading to Oxaliplatin Resistance via COL8A1-Mediated PI3K/AKT Activation in Colorectal Cancer**

**This file includes:**

**Table S1-3**

| Gene | Primer sequences |
| --- | --- |
| COL8A1 | F: AGGAAGCCGTACCCAAGAAAGG R: GGTATCCCATGACCTGGCAAAC |
| THBS2 | F: CAGTCTGAGCAAGTGTGACACC R: TTGCAGAGACGGATGCGTGTGA |

**Table S1: Detailed construction sequence of primers used in this study.**

**Table S2: Details of the ELISA kits used in this study.**

| ELISA kits | Source | Item number |
| --- | --- | --- |
| COL8A1 | ANTIBODIES ONLINE | Cat No : ABIN509884 |
| COL8A1 | ANTIBODIES ONLINE | Cat No : ABIN627617 |
| THBS2 | CUSABIO | Cat No : CSB-EL023488HU |

**Table S3: Details of the antibodies used in this study.**

| Antibodies | Source | Item number |
| --- | --- | --- |
| AKT | CST | Cat No : 9272S |
| COL8A1 | Cloud-Clone Corp | Cat No : PAC146Hu01 |
| COL8A1 | Proteintech | Cat No : 17251-1-AP |
| E-cadherin | Proteintech | Cat No : 20874-1-AP |
| FAP | Abcam | Cat No : ab207178 |
| HRP-conjugated Affinipure Goat Anti-Mouse IgG(H+L) | Proteintech | Cat No : SA00001-1 |
| HRP-conjugated Affinipure Goat Anti-Rabbit IgG(H+L) | Proteintech | Cat No : SA00001-2 |
| IgG | Beyotime | Cat No : SA00001-3 |
| ITGB1 | Affinity | Cat No : AF5379 |
| N-cadherin | Proteintech | Cat No : 22018-1-AP |
| P-AKT | CST | Cat No : 4060T |
| PI3K | Affinity | Cat No : AF6241 |
| P-PI3K | Affinity | Cat No : AF3241 |
| SNAIL | Affinity | Cat No : AF6032 |
| THBS2 | Affinity | Cat No : DF14754 |
| THBS2 | Invitrogen | Cat No : PA5-80123 |
| Vimentin | Proteintech | Cat No : 10366-1-AP |
| α-SMA | Servicebio | Cat No : GB111364 |
| β-actin | Proteintech | Cat No :66009-1-Ig |
