# Supplementary material for: THBS2 + cancer-associated fibroblasts promote EMT leading to oxaliplatin resistance via COL8A1-mediated PI3K/AKT activation in colorectal cancer
Source: Mol Cancer. 2024 Dec 28;23:282. doi: 10.1186/s12943-024-02180-y (PMC11681647; doi:10.1186/s12943-024-02180-y)
Supplement: Supplementary file 2 — Supplementary Material 2 [file 12943_2024_2180_MOESM2_ESM.docx]

**Supplementary Information for**

**THBS2+ Cancer-Associated Fibroblasts Promote EMT Leading to Oxaliplatin Resistance via COL8A1-Mediated PI3K/AKT Activation in Colorectal Cancer**

**This file includes:**

**Supplementary Methods**

**Supplementary Methods**

**Survival analysis**

The survival and survminer R packages were utilized to perform Kaplan–Meier survival analyses. The cut-off value for continuous variables in the survival data was identified by the surv_cutpoint function in the survminer R package. *p*-value was computed by the log-rank test.

**Gene set score calculation**

For bulk data, the ESTIMATE R package [1] was used to compute the immune and stromal scores and tumor purity. Gene set variation analysis (GSVA) encapsulated in the GSVA R package [2] assessed the relative pathway activity collected from The Molecular Signatures Database (MSigDB) (https://www.gsea-msigdb.org/gsea/msigdb), such as Hallmark and Reactome gene sets. Meanwhile, KEGG gene sets were obtained from the Kyoto Encyclopedia of Genes and Genomes (KEGG) (https://www.genome.jp/kegg/). The AddModuleScore function in the Seurat R package [3] scored gene sets in scRNA-seq and ST data.

**Biological enrichment analysis**

In bulk data, the limma R package [4] was used to obtain differentially expressed genes (DEGs), of which those with |log2 fold change(log2FC)| greater than 1 and false discovery rate (FDR) less than 0.05 were considered significant. KEGG analysis was performed to enrichment analysis utilizing the clusterprofiler R package [5]. Furthermore, the gene set enrichment analysis (GSEA) [6] was conducted to score Hallmark and KEGG gene sets to find the biological differences between two specific groups.

**Cell abundance estimation**

To estimate immune cell infiltration abundance of RNA data, EPIC and MCP-counter algorithms encapsulated in the IOBR R package were applied to deconvolute the proportions of various cell types [7-9]. Besides, CIBERSORTx (https://cibersortx.stanford.edu/) was utilized to estimate the abundances of cell types in bulk data using gene expression matrices from scRNA-seq data [10].

**Chemotherapeutic response assessment**

To quantify the expression similarity between distinct subgroups in two datasets, Subclass Mapping (SubMap) [11] was employed to estimate the similarity between distinct subgroups and therapeutic responders/non-responders (GSE19860) and GDSC resistant/sensitive CRC cell lines, further evaluating the potential for oxaliplatin resistance in each group.

**Identification of oxaliplatin-resistant malignant cell subpopulations**

To further explore the interaction between THBS2+ CAFs and malignant cells, the Scissor algorithm [12], which can utilize large amounts of single-cell data and phenotypic information of bulk data to identify cells that are highly correlated with phenotype, was used to identify oxaliplatin-resistant cell subpopulations. Firstly, the oxaliplatin-based chemotherapy response information in GSE19860 containing 20 non-responders and 9 responders was transformed into dichotomous variables: non-response and response. Then, we applied logistic regression in Scissor to integrate response information and malignant cluster to identify oxaliplatin-resistant cells with parameters: alpha = 0.001.

**Spatial charting of scRNA-seq**

To map the spatial coordinates in tissue, we performed an integrated analysis based on scRNA-seq and ST data through the CellTrek R package according to the tutorial [13]. The kdist function in the CellTrek package computed the spatial k-distance between different cell types with the default parameters.

**Cell-cell interactions analysis**

To infer potential communications across distinct cell types in the TME, cell-cell interactions were analyzed with default parameters using CellChat [14], which is designed for inference, analysis, and visualization of cell-cell interactions from single-cell data utilizing known molecular interaction mechanisms.

**The transcription factors (TFs) speculation with SCENIC**

To calculate the TFs of oxaliplatin-resistant malignant cells, the SCENIC algorithm was developed to speculate TFs and discover the regulon in cells [15]. Single-cell gene expression matrix with gene names in rows and cells in columns created with Seurat was imported into SCENIC. Then, the latest motif dataset was conducted to construct regulons for each TF in SCENIC. The GENIE3 constructed co-expressed genes for each TF, followed by Spearman’s correlation between the TF and the potential targets, and then generated the GRNs. Finally, AUCell analyzed regulon activity. We computed the regulon specificity score (RSS) to evaluate the cell-type specificity of a regulon.

**Trajectory analysis**

The Monocle 2 R package was applied to conduct single-cell trajectory analysis to discover the cell-state transitions, capturing the progression to the oxaliplatin-resistant phenotype [16]. The plot_cell_trajectory function was used to plot the minimum spanning tree on cells. Genes associated with EMT and oxaliplatin-resistant associated TFs were visualized by the plot_pseudotime_heatmap function.

**Drug sensitivity analysis of scRNA-seq**

To validate oxaliplatin-resistant cells and filter out drug vulnerabilities in scRNA-seq, Beyondcell was conducted to estimate the Beyondcell Score (BCS) for each drug-cell pair [17]. Firstly, we generated genesets object utilizing Drug Sensitivity Signatures collection (SSc) to compute the BCS, whose output contains the normalized and scaled BSC and the Switch Points (SPs), and information concerning the parameters used for the analysis, allowing for dimensionality reduction and clustering analysis using the bcUMAP function. Then, the bcRank calculated the BCS matrix statistics, which will be visualized their prioritization by bc4Squares function. The top left and bottom right corners of the bc4Squares plot indicated the least/most sensitive compounds for these cells, respectively. The center quadrants indicated differentially insensitive or sensitive compounds for selected cells compared to the other clusters. bcSignatures function could visualize drug signatures to identify a differential susceptibility pattern between distinct subgroups.

**Cell proliferation, transwell, and wound healing assays**

Cell Counting Kit-8 (Servicebio, Hubei, China) and Cell-Light EdU Apollo567 In Vitro Kit (RiboBio, Guangzhou, China) assays were utilized to detect the viability of HCT116 and SW480 cells according to the manufacturer’s instructions.

After processing, 2 × 10^4^ HCT116 and SW480 cells were seeded into the upper 24-transwell inserts without Matrigel (Corning, NY, USA), and 500 μl complete medium was supplied to the bottom chambers (Corning, NY, USA) for migration assays. For invasion assays, 4 × 10^4^ transfected HCT116 and SW480 cells were seeded into the upper chambers with Matrigel (Corning, NY, USA). After 48 h, the cells on the compartment were fixed in 4% paraformaldehyde (Beyotime, Shanghai, China), followed by staining with crystal violet (Solarbio, Beijing, China), then captured and quantified with an optical microscope (Cewei, Shanghai, China).

For wound healing assay, the treated cells and the NC were cultured in a medium containing 10% FBS in 6-well plates. Once the cells reached 100% confluence, a 200 µl pipette tip was subsequently applied to scratch the cell monolayer. Cells were washed twice with PBS and then captured under a phase contrast microscope immediately (0 hours) and 48 hours after wounding.

**Primary CAF isolation**Colon cancer tissue samples were collected from patients at the First Affiliated Hospital of Zhengzhou University. Additionally, CAFs were isolated from colon cancer tissues of Thbs2 conditional knockout mice to support comparative analysis. Human and mouse tissues, free of contaminants, were thoroughly washed with 5× trypsin-PBS solution (Servicebio, Wuhan, China) until the PBS clarified, removing any fat or necrotic matter. The cleaned tissues were then finely cut into 1 mm³ fragments and digested with 1 mg/ml type IV collagenase (Thermo Scientific, Shanghai, China) at 37°C for 2 hours. Following centrifugation to discard the supernatant and filtration through a 200-mesh filter, the tissues were resuspended in high-glucose DMEM containing 10% fetal bovine serum and seeded into 6 cm culture dishes. After 72 hours, the medium was replaced to remove non-adherent cells.

**Lentivirus Construction and Stable Cell Line Generation**The full-length human THBS2 gene was subcloned into the pLent-EF1a-FH-CMV-RFP-Puro lentiviral vector and packaged into pLent-THBS2 viral particles, with the empty pLent vector used as a control. Cells were infected with either pLent-THBS2 or pLent-empty viral particles and selected with puromycin for one week to establish a CAF line with stable THBS2 overexpression. The resulting stably overexpressing THBS2 (CAF-THBS2) and control (CAF-NC) cells were used for experiments within 20 passages.

**RNA isolation, reverse transcription, and qPCR**

Total RNA was extracted using TRIzon reagent (CWbiotech, Jiangsu, China), and cDNA was amplified with a HiFiScript cDNA Synthesis Kit (CWbiotech, Jiangsu, China). Real-time PCR was performed in a LineGene 9600 Plus Real time PCR System (Bioer, Hangzhou, China) using RapidStart Universal SYBR Green qPCR Mix (Monad, Suzhou, China) and the relative mRNA expression levels were determined by the cycle threshold (Ct) normalized against GAPDH levels with 2^−ΔΔCt^. For the detailed construction sequences of primers, please refer to Table S1.

**ELISA**
The culture medium was aspirated, and the extracellular levels of THBS2 and COL8A1 were measured using a human THBS2 ELISA kit, a human COL8A1 ELISA kit, and a mouse COL8A1 ELISA kit, according to the manufacturer's instructions. Results were expressed as ng/ml or pg/ml. Details of the ELISA kits used can be found in Table S2.

**Western Blot Analysis**

Tumor tissues from animals or cells cultured under specific protein or medium conditions were harvested and lysed on ice for 30 minutes using RIPA lysis buffer containing protease inhibitors (Beyotime, P1082). The lysates were then centrifuged at 12,000 rpm for 20 minutes at 4°C to obtain cell extracts. Total protein (30 μg) was separated on either 8% or 4-20% gradient SDS-PAGE gels and transferred to a PVDF membrane (Millipore, IPVH00010). The membrane was blocked for 20 minutes using a quick blocking solution and then incubated with primary antibodies at room temperature for one hour. Proteins were detected using HRP-conjugated secondary antibodies and visualized under a Tanon chemiluminescence and fluorescence imaging system (Tanon, Tanon5200) with Super ECL Plus (UElandy, S6009M). Details of the antibodies used can be found in Table S3.

**Animal models**

Four-week-old male athymic BALB/c nude mice were obtained from Vital River Laboratories (Beijing, China). All animal procedures were reviewed and approved by the Institutional Animal Care and Use Committee of Zhengzhou University. Each mouse received a subcutaneous injection of 4×10^6^ SW480 or HCT116 cells into the right axilla. Subsequently, mice were treated weekly with intratumoral injections of rhCOL8A1, oxaliplatin, or other agents based on experimental group assignments. Alternatively, each mouse received a subcutaneous injection into the right axilla of a mixture containing 3 × 10^6^ SW480 or HCT116 cells combined with 3 × 10^6^ CAF-NC or CAF-THBS2 cells, followed by different treatments according to the group assignments. Tumor volumes were recorded every four days and calculated using the formula: 0.5 × length × width^2^.

Thbs2^flox/flox^ mice and Col1a2-CreER mice were acquired from Cyagen Biosciences (Suzhou, China). The two strains were crossed to generate tissue-specific THBS2 knockout mice (Thbs2^flox/flox^; Col1a2-CreER). Genotyping confirmed the anticipated genetic modifications in both control (Thbs2^flox/flox^) and knockout (Thbs2^flox/flox^; Col1a2-CreER) mice. Tamoxifen (Merck, Darmstadt, Germany) was dissolved in corn oil (Aladdin, Shanghai, China) at a concentration of 10 mg/ml, and 0.1 ml was administered intraperitoneally per mouse. Experiments were performed 5 days post-injection.

For orthotopic mouse models, 1 × 10^4^ MC38 cells were implanted into the cecal wall of 6-8-week-old male mice, with anesthesia administered to minimize pain during the procedure. After tumor formation, treatments were applied as per experimental requirements. At the conclusion of the experiment, mice were humanely euthanized, and the collected tissues were examined to evaluate the therapeutic effects.

**Histopathological Examination**
For immunohistochemistry (IHC), tissue sections underwent a series of preparatory steps including deparaffinization, antigen retrieval, pre-staining treatments, and blocking prior to the application of secondary antibodies. The primary antibodies used were as follows: THBS2 (1:100, Invitrogen, Cat No. PA5-80123), α-SMA (1:500, Proteintech, Cat No. 80008-1-RR), FAP (1:250, Abcam, Cat No. ab207178), COL8A1 (1:200, Affinity, Cat No. DF8902), ITGB1 (1:200, Affinity, Cat No. AF5379), CDH1 (1:5000, Proteintech, Cat No. 20874-1-AP), CDH2 (1:2000, Proteintech, Cat No. 22018-1-AP), SNAIL (1:200, Affinity, Cat No. AF6032), VIM (1:2500, Proteintech, Cat No. 10366-1-AP), and ZEB1 (1:500, Proteintech, Cat No. 21544-1-AP). The secondary antibody used was HRP-conjugated goat anti-rabbit IgG (1:200, Servicebio, Cat No. GB21303). Detection was performed using a DAB chromogenic reagent kit (Servicebio, Wuhan, China) with Hematoxylin (Servicebio, Wuhan, China) serving as a nuclear counterstain.

**Multiplex Immunofluorescence**The multiplex immunofluorescence staining was conducted using the Alphapainter Fully Automated Staining Instrument (Alpha X Bio, Beijing, China). This procedure involved the sequential incubation with distinct primary antibodies, followed by application of corresponding secondary antibodies from the AlphaTSA Automation Multiplex IHC Kit (Alpha X Bio, Beijing, China), which were linked to specific fluorophores. After repeated cycles of antibody labeling, the sections were counterstained with DAPI to visualize nuclei. Imaging of the stained sections was performed using the ZEISS AXIOSCAN slide scanning system (ZEISS, Oberkochen, Germany). The primary antibodies utilized included THBS2 at a dilution of 1:100 (Invitrogen, Cat No. PA5-80123) and α-SMA at a dilution of 1:500 (Proteintech, Cat No. 80008-1-RR).

**Molecular docking and dynamics simulations**

A comprehensive molecular dynamics simulation was approached to explore the interaction between COL8A1 and ITGB1 proteins. Molecular docking was performed using ZDOCK software to identify the optimal complex for dynamic simulations. The simulations were conducted using Gromacs2022 with the Assisted Model Building with Energy Refinement (AMBER) 14SB force field for parametrizing the protein receptor and ligand under constant temperature and pressure conditions, with a 2 fs integration step and Linear Constraint Solver (LINCS) algorithm constraints for all hydrogen bonds. Post-simulation, trajectory analysis was performed using Visual Molecular Dynamics (VMD) and Pymol, and the binding free energy was calculated using the g_mmpbsa program for MMPBSA analysis. The output visualization was created via Xmgrace, VMD and optimization using R software.

**Co-immunoprecipitation (Co-IP) Assay**

Cell lysates are prepared in NP-40 lysis buffer containing protease inhibitors (Beyotime, P1082) and centrifuged to remove unlysed cellular debris. Anti-COL8A1 antibody (Cloud-Clone Corp, PAC146Hu01, 5μg) or normal rabbit IgG (Proteintech ,30000-0-AP, 2μg) is incubated with the lysates overnight at 4°C. Subsequently, 20μl of pre-washed magnetic beads are added to each sample and incubated at room temperature for one hour with rotation. The complexes are eluted by boiling in SDS sample buffer and analyzed by Western blot. Total cell lysates are used as input, with normal rabbit IgG serving as the negative control.

**Reference**

1. Yoshihara K, et al. Inferring tumour purity and stromal and immune cell admixture from expression data. Nat Commun. 2013;4(2612. https://doi.org/10.1038/ncomms3612.

2. Hanzelmann S, Castelo R, Guinney J. GSVA: gene set variation analysis for microarray and RNA-seq data. BMC Bioinformatics. 2013;14(7. https://doi.org/10.1186/1471-2105-14-7.

3. Hao Y, et al. Integrated analysis of multimodal single-cell data. Cell. 2021;184(13):3573-3587 e3529. https://doi.org/10.1016/j.cell.2021.04.048.

4. Ritchie ME, et al. limma powers differential expression analyses for RNA-sequencing and microarray studies. Nucleic Acids Res. 2015;43(7):e47. https://doi.org/10.1093/nar/gkv007.

5. Wu T, et al. clusterProfiler 4.0: A universal enrichment tool for interpreting omics data. Innovation (Camb). 2021;2(3):100141. https://doi.org/10.1016/j.xinn.2021.100141.

6. Subramanian A, et al. Gene set enrichment analysis: a knowledge-based approach for interpreting genome-wide expression profiles. Proc Natl Acad Sci U S A. 2005;102(43):15545-15550. https://doi.org/10.1073/pnas.0506580102.

7. Racle J, et al. Simultaneous enumeration of cancer and immune cell types from bulk tumor gene expression data. eLife. 2017;6(https://doi.org/10.7554/eLife.26476.

8. Becht E, et al. Estimating the population abundance of tissue-infiltrating immune and stromal cell populations using gene expression. Genome Biol. 2016;17(1). https://doi.org/10.1186/s13059-016-1070-5.

9. Zeng D, et al. IOBR: Multi-Omics Immuno-Oncology Biological Research to Decode Tumor Microenvironment and Signatures. Front Immunol. 2021;12(687975. https://doi.org/10.3389/fimmu.2021.687975.

10. Newman AM, et al. Determining cell type abundance and expression from bulk tissues with digital cytometry. Nat Biotechnol. 2019;37(7):773-782. https://doi.org/10.1038/s41587-019-0114-2.

11. Hofmann O, et al. Subclass Mapping: Identifying Common Subtypes in Independent Disease Data Sets. PLoS One. 2007;2(11). https://doi.org/10.1371/journal.pone.0001195.

12. Sun D, et al. Identifying phenotype-associated subpopulations by integrating bulk and single-cell sequencing data. Nat Biotechnol. 2022;40(4):527-538. https://doi.org/10.1038/s41587-021-01091-3.

13. Wei R, et al. Spatial charting of single-cell transcriptomes in tissues. Nat Biotechnol. 2022;40(8):1190-1199. https://doi.org/10.1038/s41587-022-01233-1.

14. Jin S, et al. Inference and analysis of cell-cell communication using CellChat. Nat Commun. 2021;12(1):1088. https://doi.org/10.1038/s41467-021-21246-9.

15. Aibar S, et al. SCENIC: single-cell regulatory network inference and clustering. Nature Methods. 2017;14(11):1083-1086. https://doi.org/10.1038/nmeth.4463.

16. Qiu X, et al. Reversed graph embedding resolves complex single-cell trajectories. Nat Methods. 2017;14(10):979-982. https://doi.org/10.1038/nmeth.4402.

17. Fustero-Torre C, et al. Beyondcell: targeting cancer therapeutic heterogeneity in single-cell RNA-seq data. Genome Med. 2021;13(1):187. https://doi.org/10.1186/s13073-021-01001-x.
