# Supplementary material for: THBS2 + cancer-associated fibroblasts promote EMT leading to oxaliplatin resistance via COL8A1-mediated PI3K/AKT activation in colorectal cancer
Source: Mol Cancer. 2024 Dec 28;23:282. doi: 10.1186/s12943-024-02180-y (PMC11681647; doi:10.1186/s12943-024-02180-y)
Supplement: Supplementary file 3 — Supplementary Material 3 [file 12943_2024_2180_MOESM3_ESM.docx]

**Supplementary Information for**

**THBS2+ Cancer-Associated Fibroblasts Promote EMT Leading to Oxaliplatin Resistance via COL8A1-Mediated PI3K/AKT Activation in Colorectal Cancer**

**This file includes:**

**Figures S1–S10**

**Supplemental Figures**

**
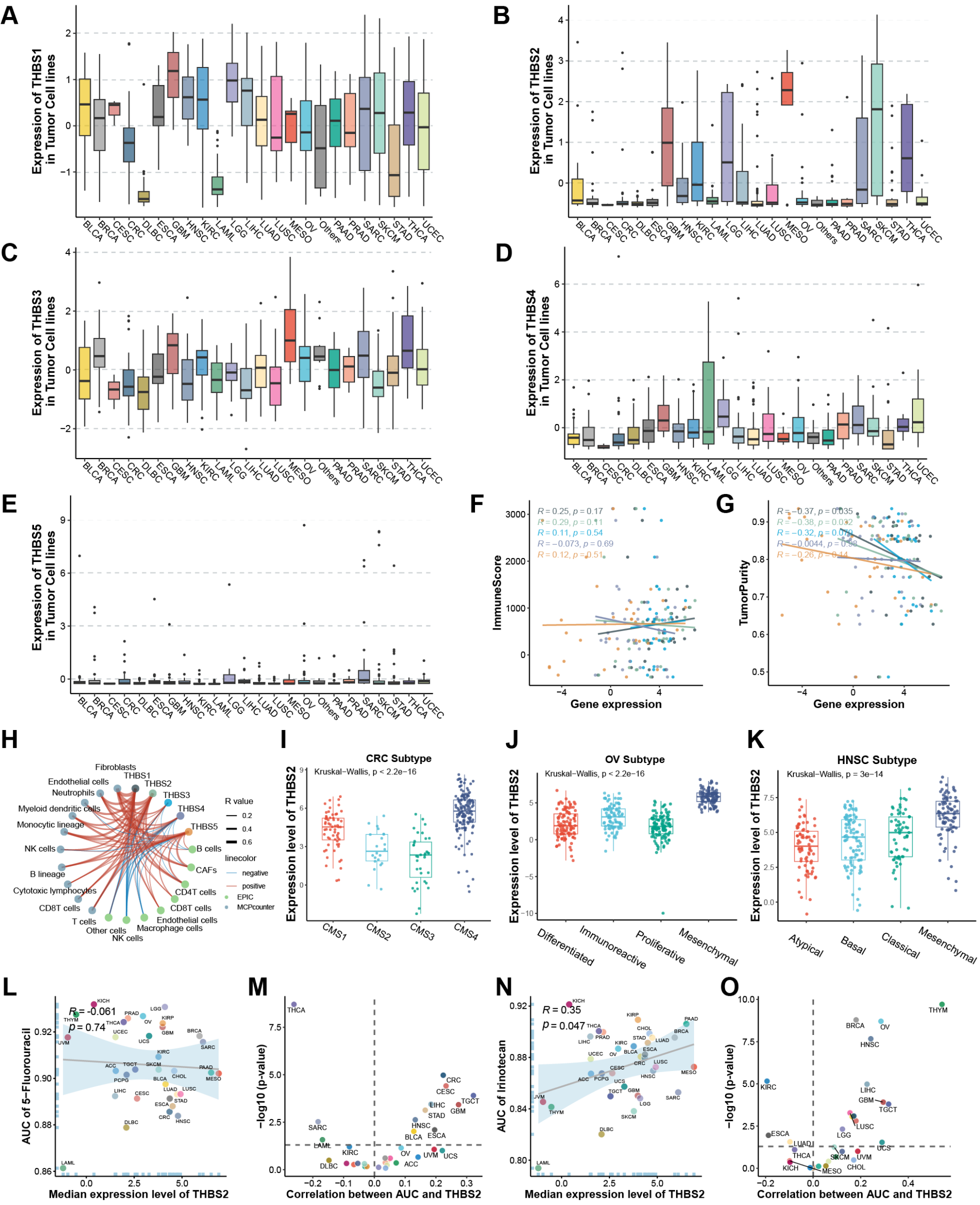
**

**Figure S1.** **THBS2 was associated with EMT phenotype and chemoresistance.** (A-E) THBS family mRNA expression levels across various cancer cell lines. (F-G) The Spearman correlation of THBS family mRNA expression and immune score and tumor purity calculated by ESTIMATE algorithm in TCGA, respectively. (H) The Spearman correlation of THBS2 mRNA expression and immune and stromal cells abundance estimated by MCP-counter and EPIC algorithms in TCGA. (I-K) The difference in the THBS2 mRNA expression among distinct subtypes of CRC, OV, and HNSC, respectively. (L) The Spearman correlation of median THBS2 mRNA expression and median AUC of 5-fluorouracil across the cancer types. (M) The Spearman correlation of THBS2 mRNA expression and AUC of 5-fluorouracil for each cancer type. (N) The Spearman correlation of median THBS2 mRNA expression and median AUC of irinotecan across the cancer types. (O) The Spearman correlation of THBS2 mRNA expression and AUC of irinotecan for each cancer type.


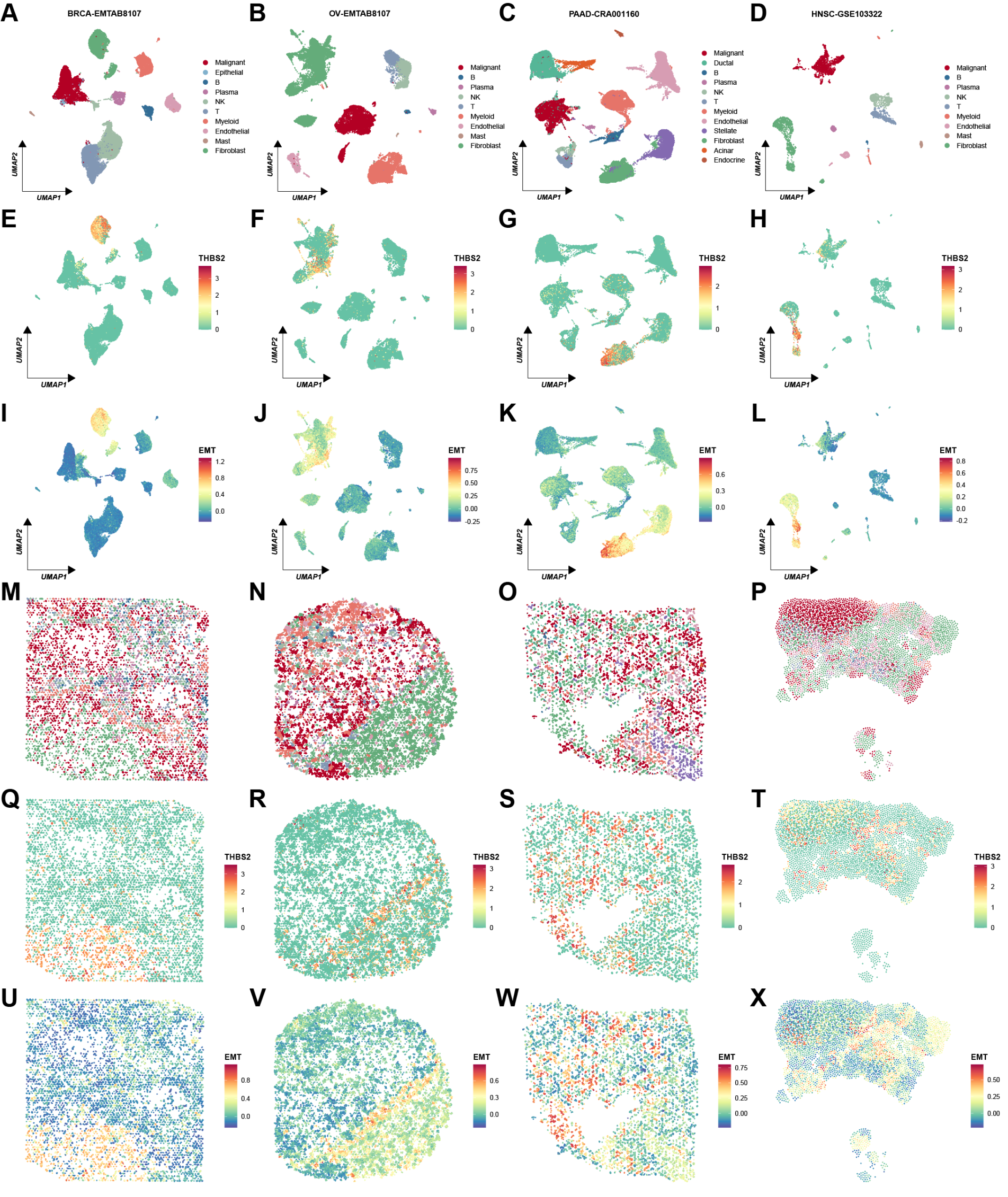


**Figure S2. THBS2 facilitated the CAF activation and EMT phenotype.** (A-D) UMAP plot of the identified cell types. Different colors represented the different cell types. (E-H) UMAP plot of the identified cells colored by THBS2 expression. (I-L) UMAP plot of the identified cells colored by EMT score calculated by AddMouduleScore function. (M-P) Assignment of cell subtypes and their spatial distributions using CellTrek algorithm. (Q-T) The expression of THBS2 based on ST analysis. (U-X) The EMT score was calculated by AddMouduleScore based on ST analysis.


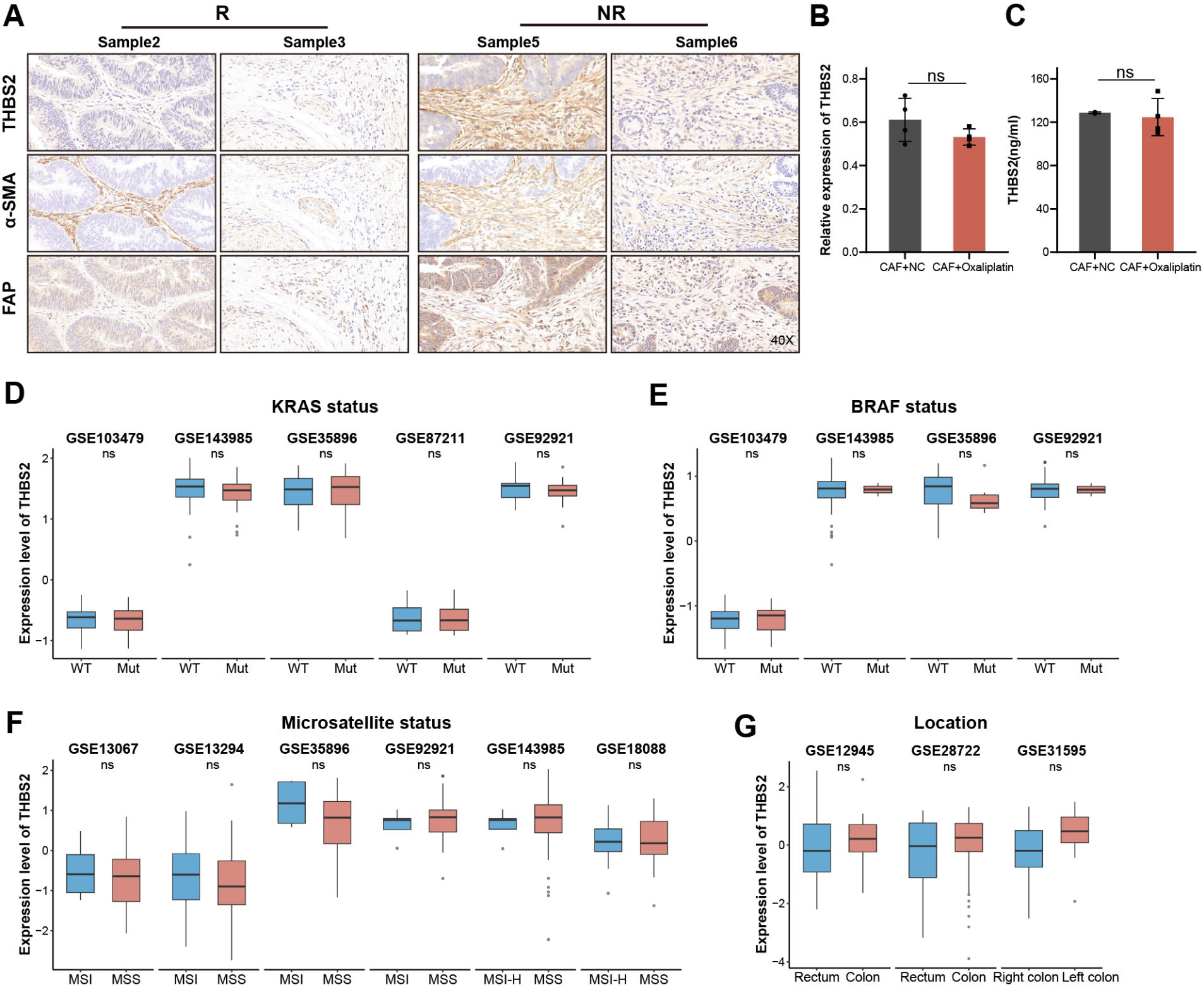


**Figure S3. THBS2 contributed to oxaliplatin therapeutic resistance in CRC.** (A) Representative images of IHC staining for THBS2, α-SMA and FAP in responder (R) group and non-responder (NR) group. (B) qPCR analysis of THBS2 mRNA level in CAF treated with NC or oxaliplatin. (C) ELISA quantification of THBS2 level in the supernatant of CAF treated with NC or oxaliplatin. (D-G) The difference in THBS2 expression among KRAS, BRAF, microsatellite status and anatomical location in multiple CRC datasets, respectively. ns, not significant.


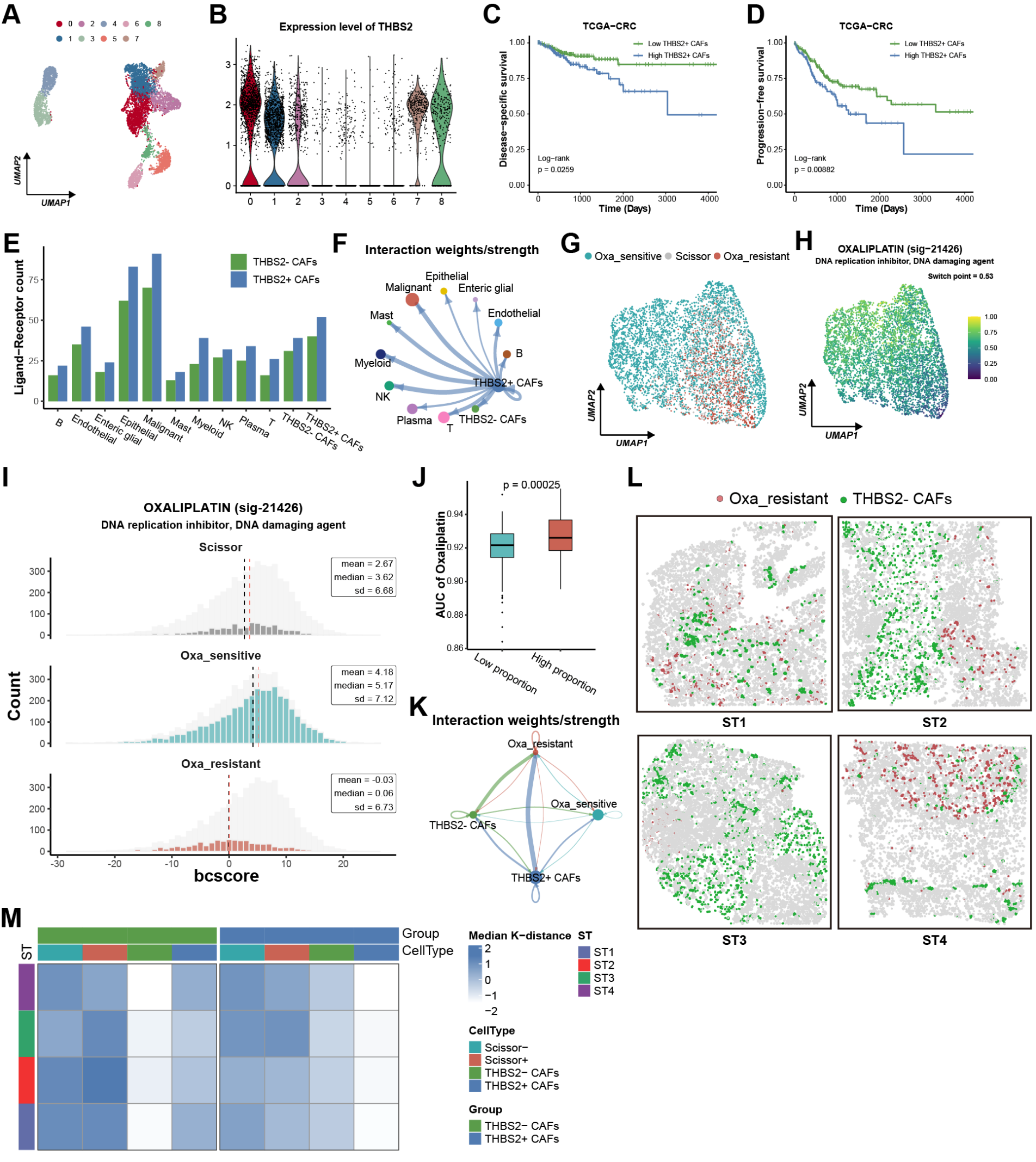


**Figure S4. THBS2+ CAFs were associated with oxaliplatin resistance in CRC.** (A) UMAP plot of the identified different CAF subclusters. Different colors represented the different subclusters. (B) Violin plot showing THBS2 expression across different CAF subclusters. (C-D) Kaplan-Meier curve of DSS and PFS between high and low THBS2+ CAFs abundance deconvoluted by CIBERSORTx algorithm in TGCA-CRC, respectively. (E) The counts of ligand-receptor interactions from CAFs to other cell types inferred by CellChat algorithm. (F) The difference in AUC of oxaliplatin between high and low oxaliplatin-resistant abundance groups deconvoluted by CIBERSORTx algorithm. The oxaliplatin AUC value for each sample in TCGA-CRC was predicted by pRRophetic algorithm. (G) UMAP plot of the identified different malignant subtypes defined by Beyondcell algorithm. Different colors represented the different subtypes. (H) UMAP plot of Beyondcell score of oxaliplatin calculated by Beyondcell algorithm in different malignant subtypes. (I) Histogram representing Beyondcell score of oxaliplatin calculated by Beyondcell algorithm for each malignant subtype. (J) Spatial cell charting of oxaliplatin-resistant cells and THBS2- CAFs using CellTrek algorithm. (K) Heat map representing the spatial distance from CAFs subtypes to malignant cell subtypes.


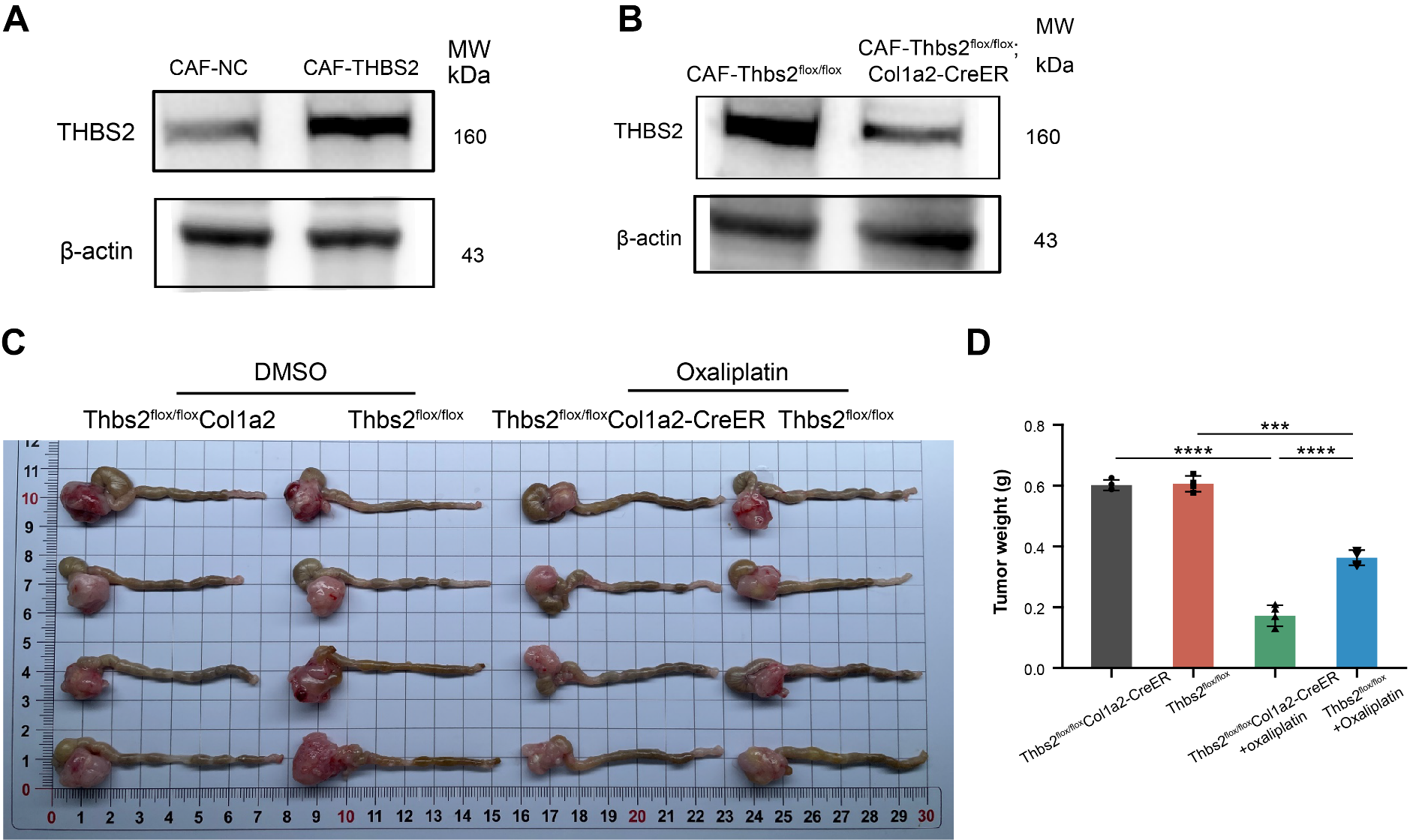


**Figure S5. Fibroblast-specific Thbs2 knockout mice revealed the impact of THBS2 on oxaliplatin resistance.** (A) Western blotting demonstrating the overexpression efficiency of THBS2 in transfected CAF cells (CAF-THBS2). (B) Western blotting demonstrating the knockout efficiency of THBS2 in fibroblast-specific Thbs2 knockout mice (Thbs2^flox/flox^; Col1a2-CreER). (C-D) Representative images of fibroblast-specific Thbs2 knockout mice (Thbs2^flox/flox^; Col1a2-CreER) treated with DMSO or oxaliplatin. Tumor weight was measured after implantation. ****p* < 0.001, *****p* < 0.0001.

**
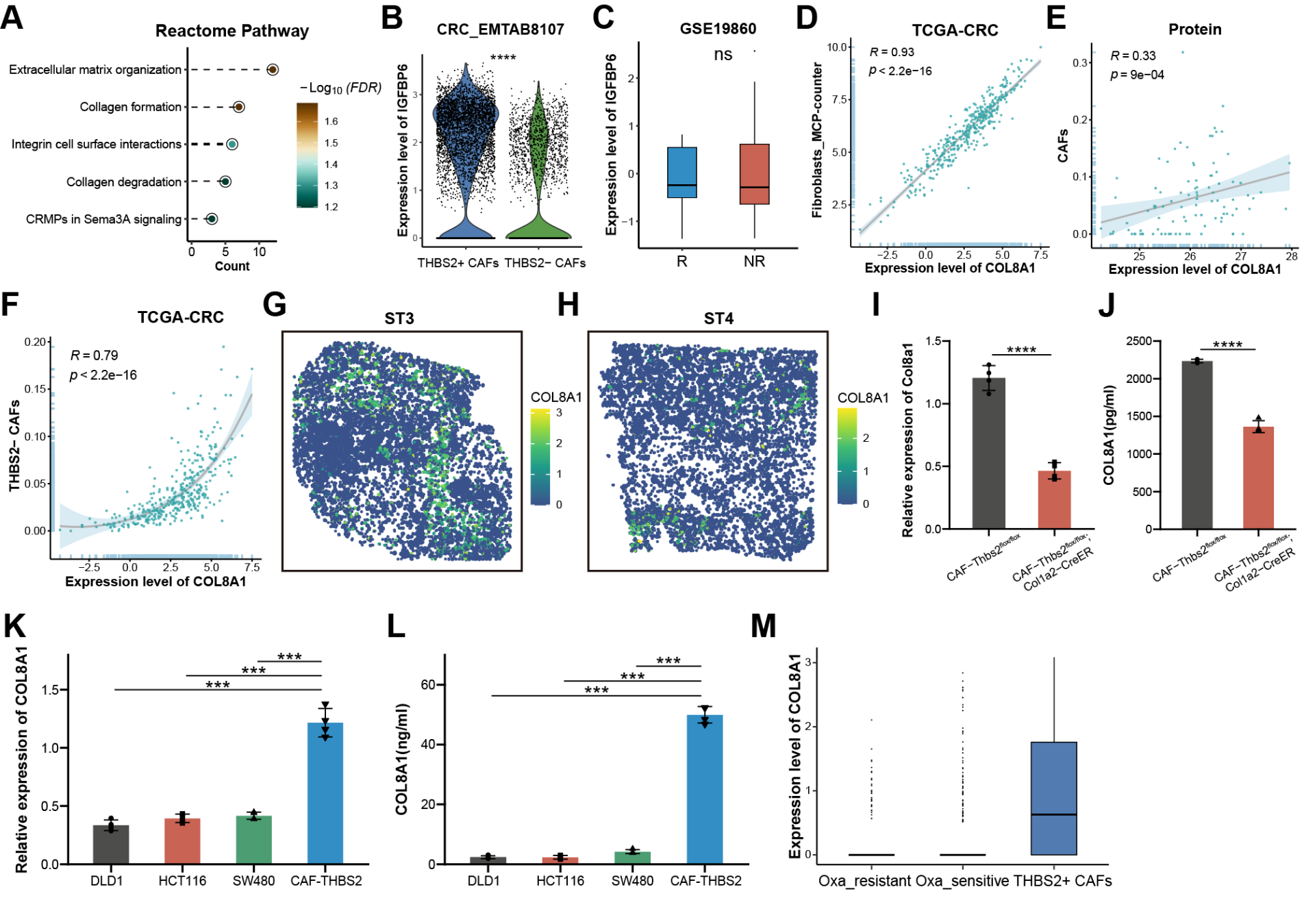
**

**Figure S6. COL8A1 derived from THBS2+ CAFs facilitated oxaliplatin resistance in CRC.** (A) Functional enrichment analysis of upregulated DEGs between resistant cells and sensitive cells in GSE42387. (B) Violin plot showing IGFBP6 mRNA expression across different CAF subtypes. (C) The difference in IGFBP6 mRNA expression between the non-responder (NR) and the responder (R) group in GSE19860. (D) The Spearman correlation of COL8A1 mRNA expression and CAFs abundance deconvoluted by MCP-counter algorithm in TCGA-CRC. (E) The Spearman correlation of COL8A1 protein expression and CAFs abundance derived from CPTAC database. (F) The Spearman correlation of COL8A1 mRNA expression and THBS2- CAFs abundance deconvoluted by CIBERSORTx algorithm in TCGA-CRC. (G-H) The mRNA expression of COL8A1 based on ST analysis. (I) qPCR analysis of Col8a1 mRNA levels in fibroblast-specific Thbs2 knockout mice (Thbs2^flox/flox^; Col1a2-CreER) and control (Thbs2^flox/flox^). (J) ELISA quantification of COL8A1 levels in the supernatant of fibroblast-specific Thbs2 knockout mice (Thbs2^flox/flox^; Col1a2-CreER) and control (Thbs2^flox/flox^). (K) qPCR analysis of COL8A1 mRNA level in three CRC cell lines and CAF-THBS2. (L) ELISA quantification of COL8A1 level in the supernatant of three CRC cell lines and CAF-THBS2. (M) scRNA-seq analysis showing the COL8A1 expression level among oxaliplatin resistant and sensitive malignant cells as well as THBS2+ CAFs. ns, not significant, ****p* < 0.001, *****p* < 0.0001


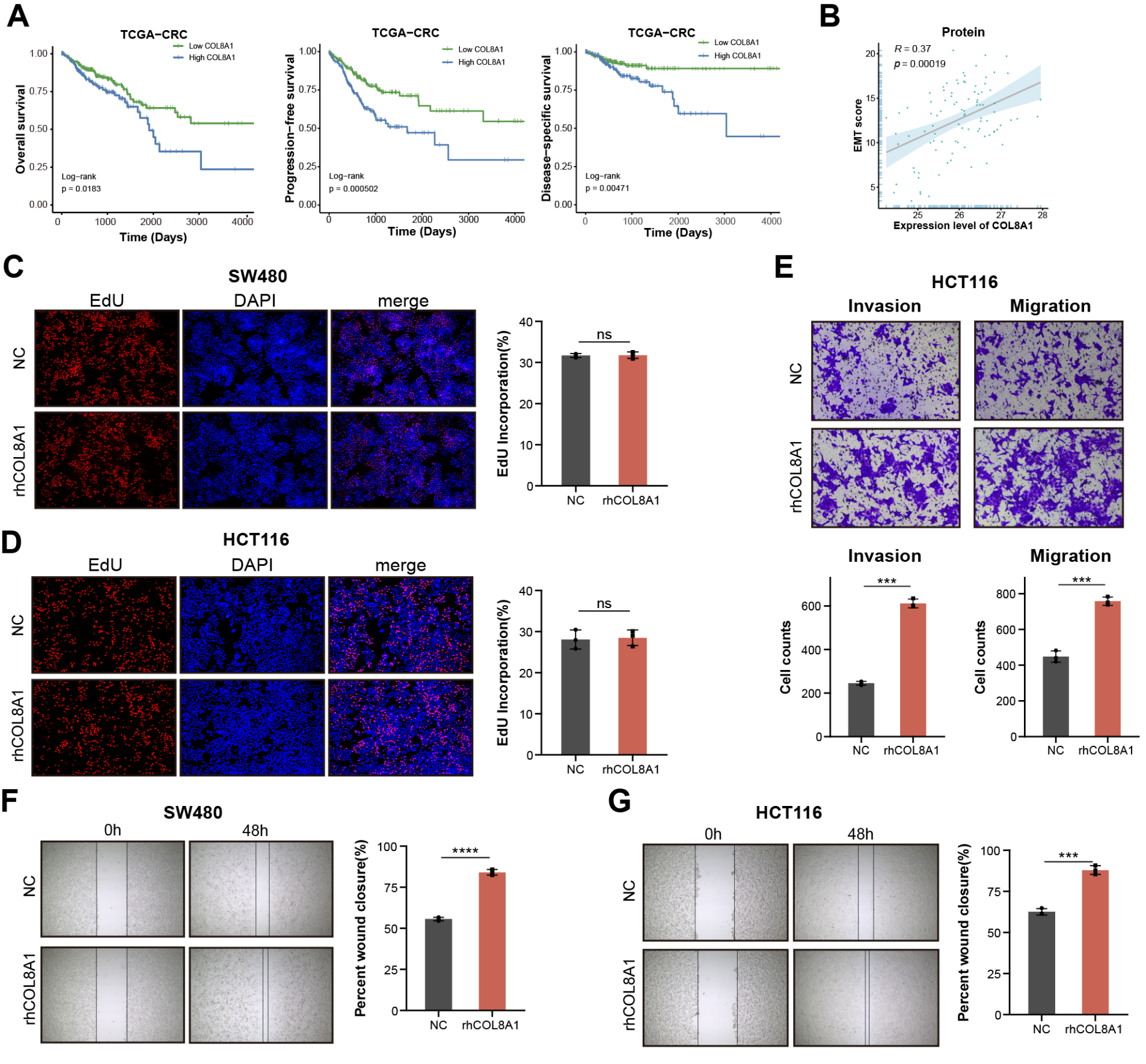


**Figure S7. COL8A1 had a positive connection to EMT in CRC.** (A) Kaplan-Meier curves of OS, DSS and PFS between high and low COL8A1 expression in TGCA-CRC using the log-rank test, respectively. (B) The Spearman correlation of COL8A1 protein expression and EMT score derived from CPTAC. (C-D) Representative data from EdU assays performed in CRC cells treated with rhCOL8A1 or NC. (E) Representative data from migration assays performed in HCT116 cells treated with rhCOL8A1 or NC. (F-G) Representative data from wound healing assays performed in CRC cells treated with rhCOL8A1 or NC. ns, not significant, ****p* < 0.001, *****p* < 0.0001


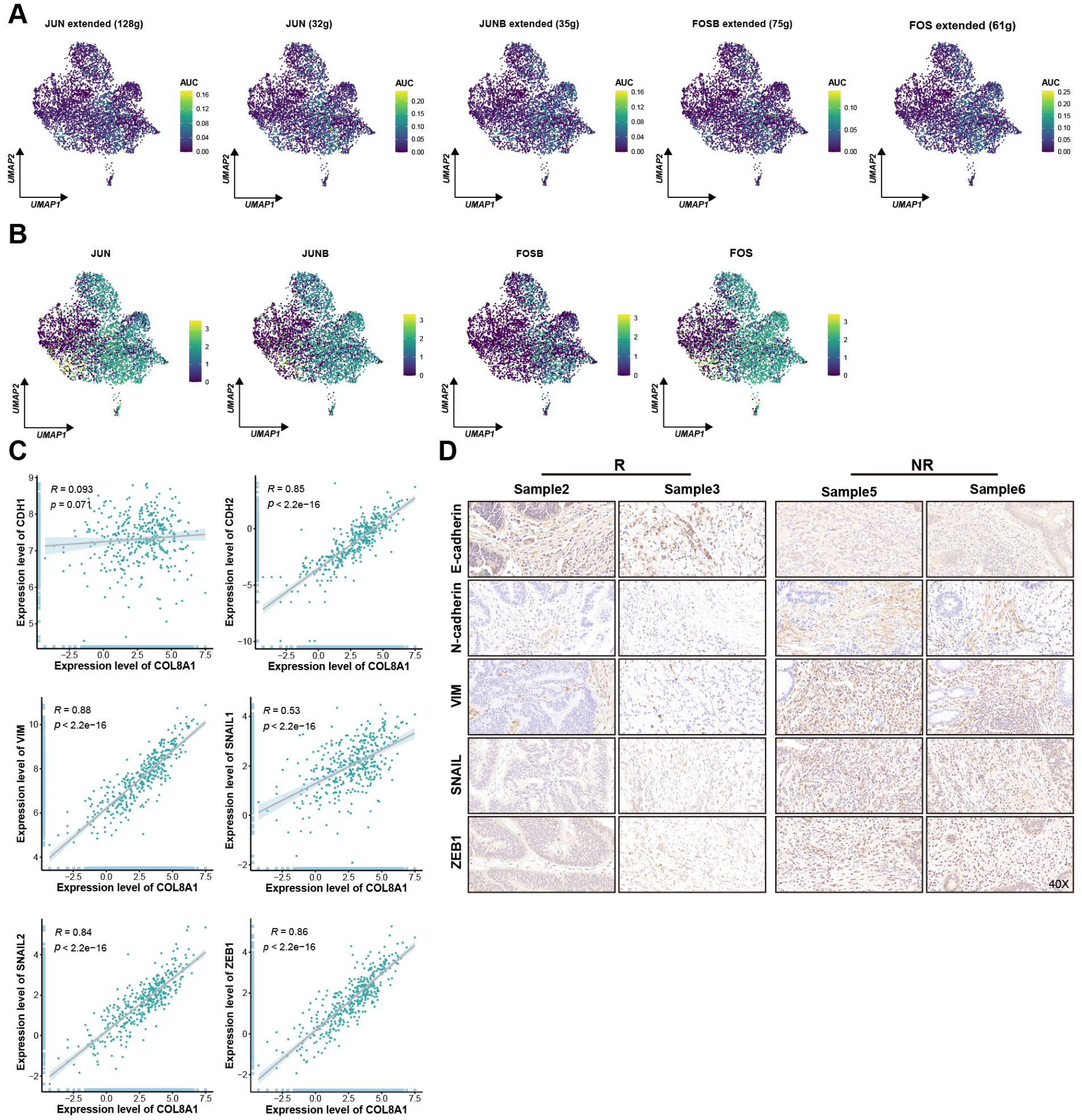


**Figure S8. COL8A1 activated EMT leading to oxaliplatin resistance in CRC.** (A) UMAP plot of AUC values of the TFs JUN_extented (128g), JUN (32g), JUNB_extented (35g), FOSB_extented (75g) and FOS_extented (61g) in oxaliplatin-resistant cells, respectively. (B) UMAP plot of FOS, JUN, JUNB, and FOSB expression in malignant cells, respectively. (C) The Spearman correlation of COL8A1 expression and E-cadherin (CDH1), SNAIL2, ZEB1 and VIM in TCGA-CRC, respectively. (D) Representative images of IHC staining for E-cadherin, N-cadherin, VIM, ZEB1 and SNAIL in responder (R) group and non-responder (NR) group.

**
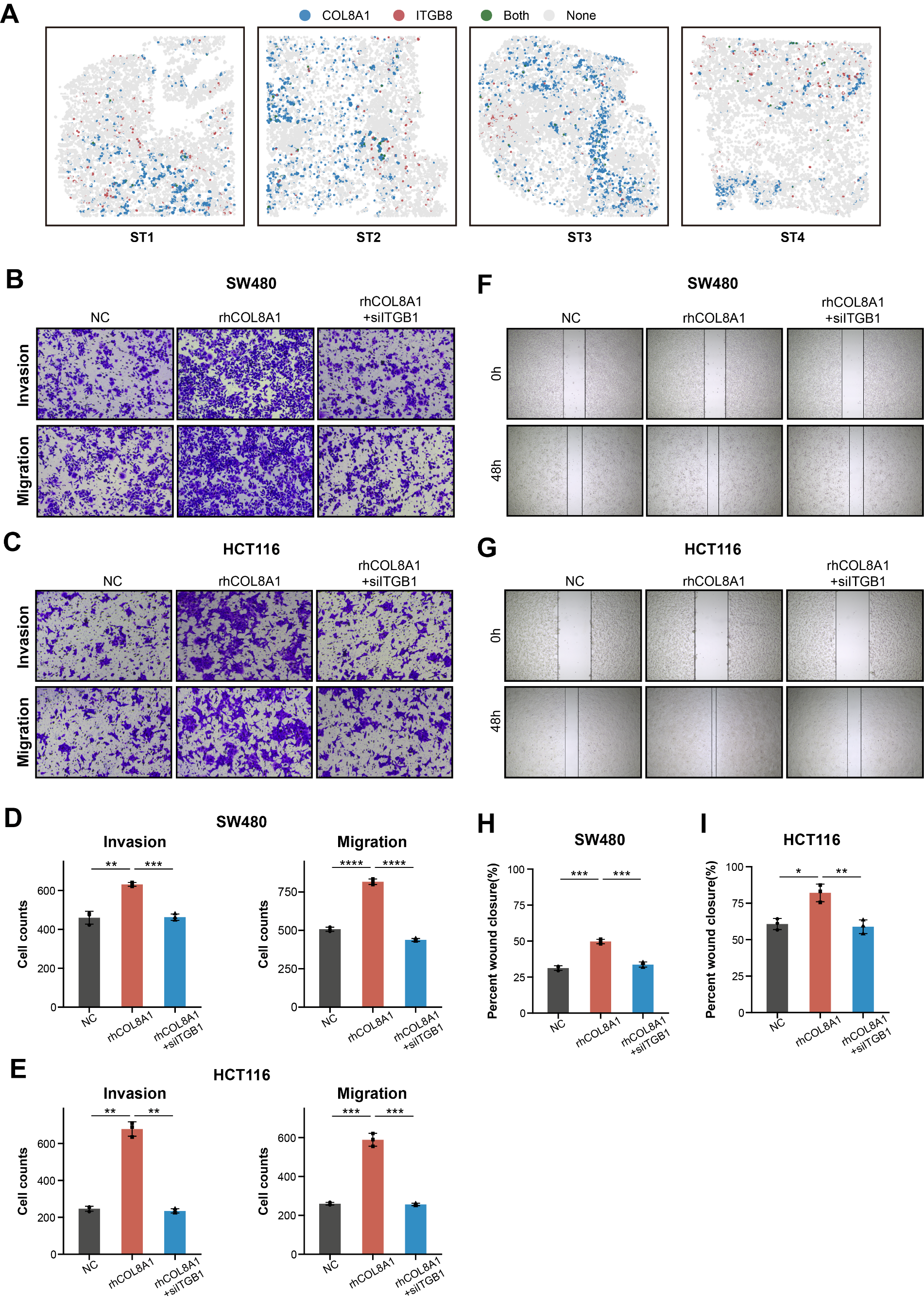
**

**Figure S9. COL8A1 interacting with ITGB1 contributed to oxaliplatin resistance.** (A) Visualization of COL8A1-ITGB8 interaction of ST data. (B-E) Representative data from invasion and migration assays performed in CRC cells treated with rhCOL8A1 or combined with siITGB1. (F-I) Representative data from wound healing assays performed in HCT116 cells treated with rhCOL8A1 or combined with siITGB1. ***p* < 0.01, ****p* < 0.001, *****p* < 0.0001


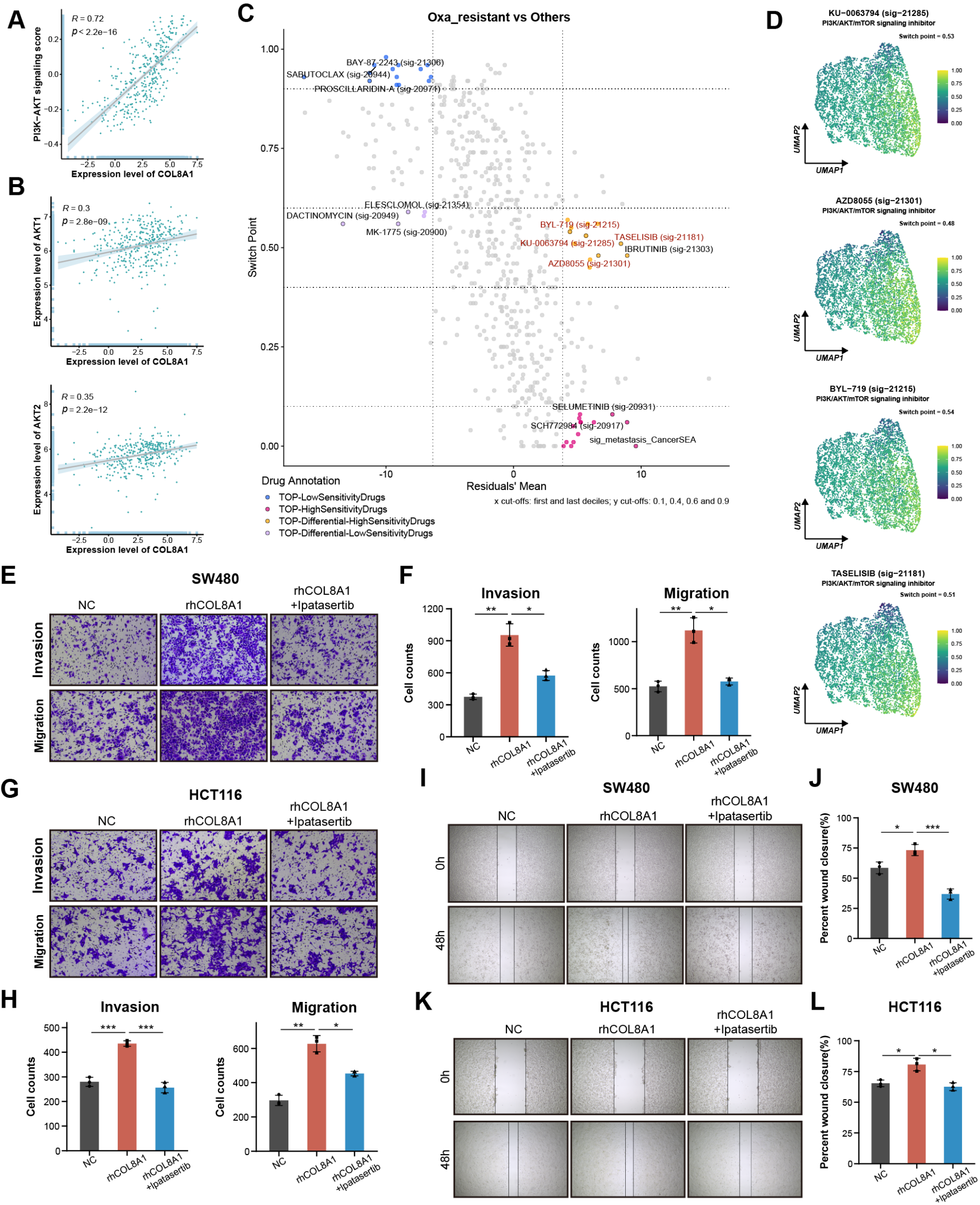


**Figure S10. COL8A1 interfered with PI3K-AKT signaling promoting oxaliplatin resistance.** (A) The Spearman correlation of COL8A1 expression and PI3K-AKT pathway score calculated by GSVA in TCGA-CRC. (B) The Spearman correlation of COL8A1 expression and AKT1 and AKT2 expression in TCGA-CRC, respectively. (C) Summary of Beyondcell’s proposed drugs to target oxaliplatin-resistant cells. (D) UMAP plot of Beyondcell scores of KU-0063794, AZD8055, BYL-719, and taselisib calculated by the Beyondcell algorithm, respectively. (E-H) Representative data from migration assays performed in CRC cells treated with rhCOL8A1 or combined with Ipatasertib. (I-L) Representative data from wound healing assays performed in CRC cells treated with rhCOL8A1 or combined with Ipatasertib. **p* < 0.05, ***p* < 0.01, ****p* < 0.001, *****p* < 0.0001
